# Genome-Wide Association Study Implicates Testis-Sperm Specific *FKBP6* as a Susceptibility Locus for Impaired Acrosome Reaction in Stallions

**DOI:** 10.1371/journal.pgen.1003139

**Published:** 2012-12-20

**Authors:** Terje Raudsepp, Molly E. McCue, Pranab J. Das, Lauren Dobson, Monika Vishnoi, Krista L. Fritz, Robert Schaefer, Aaron K. Rendahl, James N. Derr, Charles C. Love, Dickson D. Varner, Bhanu P. Chowdhary

**Affiliations:** 1 Department of Veterinary Integrative Biosciences, Texas A&M University, College Station, Texas, United States of America; 2 Department of Veterinary Population Medicine, University of Minnesota, St. Paul, Minnesota, United States of America; 3 Department of Veterinary Pathobiology, Texas A&M University, College Station, Texas, United States of America; 4 Department of Veterinary and Biochemical Sciences, University of Minnesota, St. Paul, Minnesota, United States of America; 5 School of Statistics, University of Minnesota, Minneapolis, Minnesota, United States of America; 6 Department of Large Animal Clinical Sciences, Texas A&M University, College Station, Texas, United States of America; Stanford University School of Medicine, United States of America

## Abstract

Impaired acrosomal reaction (IAR) of sperm causes male subfertility in humans and animals. Despite compelling evidence about the genetic control over acrosome biogenesis and function, the genomics of IAR is as yet poorly understood, providing no molecular tools for diagnostics. Here we conducted Equine SNP50 Beadchip genotyping and GWAS using 7 IAR–affected and 37 control Thoroughbred stallions. A significant (*P*<6.75E-08) genotype–phenotype association was found in horse chromosome 13 in FK506 binding protein 6 (*FKBP6*). The gene belongs to the immunophilins FKBP family known to be involved in meiosis, calcium homeostasis, clathrin-coated vesicles, and membrane fusions. Direct sequencing of *FKBP6* exons in cases and controls identified SNPs g.11040315G>A and g.11040379C>A (p.166H>N) in exon 4 that were significantly associated with the IAR phenotype both in the GWAS cohort (n = 44) and in a large multi-breed cohort of 265 horses. All IAR stallions were homozygous for the A-alleles, while this genotype was found only in 2% of controls. The equine *FKBP6* was exclusively expressed in testis and sperm and had 5 different transcripts, of which 4 were novel. The expression of this gene in AC/AG heterozygous controls was monoallelic, and we observed a tendency for *FKBP6* up-regulation in IAR stallions compared to controls. Because exon 4 SNPs had no effect on the protein structure, it is likely that *FKBP6* relates to the IAR phenotype via regulatory or modifying functions. In conclusion, *FKBP6* was considered a susceptibility gene of incomplete penetrance for IAR in stallions and a candidate gene for male subfertility in mammals. *FKBP6* genotyping is recommended for the detection of IAR–susceptible individuals among potential breeding stallions. Successful use of sperm as a source of DNA and RNA propagates non-invasive sample procurement for fertility genomics in animals and humans.

## Author Summary

Impaired acrosomal reaction (IAR) of sperm causes male subfertility in humans and animals, and currently the molecular causes of the condition are not known. Here we report the mapping, identification, and functional analysis of a susceptibility locus for IAR in stallions. The candidate region was mapped to horse chromosome 13 by SNP genotyping and GWAS of 7 IAR affected and 44 control Thoroughbred stallions. Re-sequencing and case-control analysis of functionally relevant candidate genes in the region identified *FKBP6* gene as a significantly associated locus. The association was confirmed by genotyping 265 male horses of multiple breeds. *FKBP6* belongs to the immunophilins FKBP family known to be involved in meiosis, calcium homeostasis, clathrin-coated vesicles, and membrane fusions. We showed that the equine *FKBP6* is exclusively and monoallelically expressed in testis and sperm and has 5 different transcripts, of which 4 were novel. Overall, *FKBP6* was considered a susceptibility gene of incomplete penetrance for IAR in stallions and a candidate gene for male subfertility in other mammals. Successful use of sperm as a source of DNA and RNA propagates non-invasive sample procurement for fertility genomics in animals and humans.

## Introduction

Acrosome is a large, membrane bound secretory vesicle in the head of mammalian sperm which releases its contents when sperm contacts the egg for fertilization. The process is known as regulated acrosomal exocytosis or acrosomal reaction (AR) and is triggered by egg's zona pellucida proteins which induce an influx of calcium in to the sperm. Acrosomal exocytosis facilitates the passage of the sperm through the zona and is obligatory for natural fertilization [1], [2], [3], [4], [5]. The AR is a one-time event during which the acrosome releases all its contents and loses structural integrity [2]. Consequently, any kind of acrosome dysfunction, *i.e.*, premature AR, reduced release of the acrosomal contents or the absence of AR, contribute to male subfertility because such sperm are incapable of fertilization [1], [6].

The success of AR is determined by a number of events such as acrosome biogenesis, sperm capacitation, sperm-oocyte recognition, sperm-zona binding, and elicitation of signaling pathways. Each of these and the AR are regulated by multiple proteins and protein complexes [1], [2], [5], [7], [8], the best known of which is SNARE – a group of proteins that controls membrane fusion and exocytosis in humans and mice, both in the sperm acrosome [7], [9] and neuronal synapses [10].

Current knowledge about the genetic regulation of AR is limited. Knockout and mutant rodent models have revealed 32 genotypes and 26 genes that are associated with impaired AR (IAR) phenotype (MGI: http://www.informatics.jax.org/). The AR involves genes for neurotransmitter receptors [11], [12], calcium channels [13], [14], [15], membrane fusions [16] and vesicle exocytosis [9], [17]. Mouse triple knockout models for five sperm motility and sperm-egg adhesion genes (*Tnp2*, *H1.1*, *H1t*, *Smcp*) suggest that AR might be the result of synergistic interaction of several genes [18]. The few mutations associated with IAR have been found only in mouse [11], [12], while the molecular causes of IAR in humans and other non-rodent mammals remain as yet poorly understood. At the same time, it has been proposed that about 20–25% of cases of unexplained male subfertility in humans can be attributed to defects in sperm functions responsible for fertilization - the AR and sperm's ability to bind and penetrate the zona [7], [19], [20], [21], [22], [23]. The prevalence of these conditions in horses is not known but the incidence of unexplained subfertility in breeding stallions mimics that of humans [24], [25]. Recently, a few such cases in Thoroughbreds were associated with IAR [25].

Stallion fertility is one of the key components of the economy of the equine industry. Despite this, to date, with the exception of a few chromosome aberrations [26] and Y chromosome deletions [27], no genetic mutations have been associated with reproductive disorders in stallions. This is likely because the genetic regulation of male fertility and reproduction in mammals is thought to involve 10–20% of the genes in the genome [28], [29], [30], [31] which complicates the candidate gene approach for mutation discovery and requires the use of pan-genomic platforms, such as SNP chips that facilitate hypothesis free, genome-wide discovery of associated genes or genomic regions.

Here we report about a Genome Wide Association Study (GWAS) that resulted in the mapping, identification and functional analysis of a susceptibility locus for impaired acrosome reaction in stallions.

## Results

### Relatedness and stratification within the study population

Analysis of five generation pedigrees in the 44 horses used for SNP genotyping showed moderate to low degree of relatedness among the seven affected Thoroughbred stallions (Table 1). Six animals (HS03, HS29, HS30, HS32, HS33, HS34) formed one closely related group, with relatedness coefficient (r) values between 11 and 30%, and shared common ancestors in paternal, maternal or in both parental lineages. The percentage of genes shared by common descent was the highest (r = 30.6%) between stallions HS30 and HS34 (Table 1). One of the affected stallions (HS31) clearly originated from a different Thoroughbred lineage with r<8% to other animals. In contrast to the moderate relatedness values, inbreeding coefficients for the seven stallions were relatively low and ranged from 2.2% to 4.3% implying low genetic relationships among the parents of the affected individuals. A detailed, five-generation pedigree for the 7 cases and 37 control male Thoroughbreds is presented in Figure S1.

**Table 1:** Inbreeding coefficients (diagonal boxes) and genetic relationship coefficients of and among the seven affected stallions calculated from i) pedigree data in bold (upper triangle of the matrix) and from ii) SNP genotyping data in normal font (lower triangle of the matrix).

	Stallion HS30	Stallion HS31	Stallion HS34	Stallion HS32	Stallion HS03	Stallion HS29	Stallion HS33
Stallion HS30	* ***2.9%***	**0.064**	**0.306**	**0.194**	**0.172**	**0.188**	**0.184**
Stallion HS31	0.014	* ***4.0%***	**0.08**	**0.056**	**0.056**	**0.058**	**0.060**
Stallion HS34	0.331	0.088	* ***4.2%***	**0.202**	**0.176**	**0.192**	**0.194**
Stallion HS32	0.130	0.000	0.194	* ***4.3%***	**0.114**	**0.134**	**0.130**
Stallion HS03	0.164	0.062	0.214	0.097	* ***2.2%***	**0.296**	**0.116**
Stallion HS29	0.149	0.019	0.179	0.093	0.265	* ***3.7%***	**0.126**
Stallion HS33	0.143	0.038	0.184	0.065	0.090	0.109	* ***3.3%***

Stallion code names correspond to the codes in the 5-generation pedigree (Figure S1).

* The diagonal boxes in ***bold italics with %*** that should correspond to self-to-self genetic relationship ( = 1) have been used to show inbreeding coefficients for each of the 7 stallions.

Similarly, calculation of relationships from genome wide SNP genotypes demonstrated a greater extent of genome sharing among the 7 cases, with estimates of genome sharing identity by descent (IBD) between case-case pairs (mean 0.126; standard deviation, sd 0.085) higher than estimates of genome sharing IBD between control-control pairs (mean 0.026; sd 0.049), or across the entire cohort (mean 0.032; sd 0.05). Relationship coefficients for the 7 cases were similar between the SNP and pedigree data (Table 1), with HS31 being less related to the other 6 cases. Separation of HS31 from other cases, as well as, relative clustering of cases when compared to controls was also visualized on multidimensional scaling (MDS) plots (Figure S2). Empirical *P*-values for the group differences in IBS sharing demonstrate that on average, cases were more related to other cases (*P* = 3.99E-05) than controls were related to other controls (*P* = 0.006), however, overall case-case pairs were not significantly (*P* = 0.5) more related than case-control pairs (Table S1).

### Genome-wide SNP association study (GWAS)

**Figure 1:**
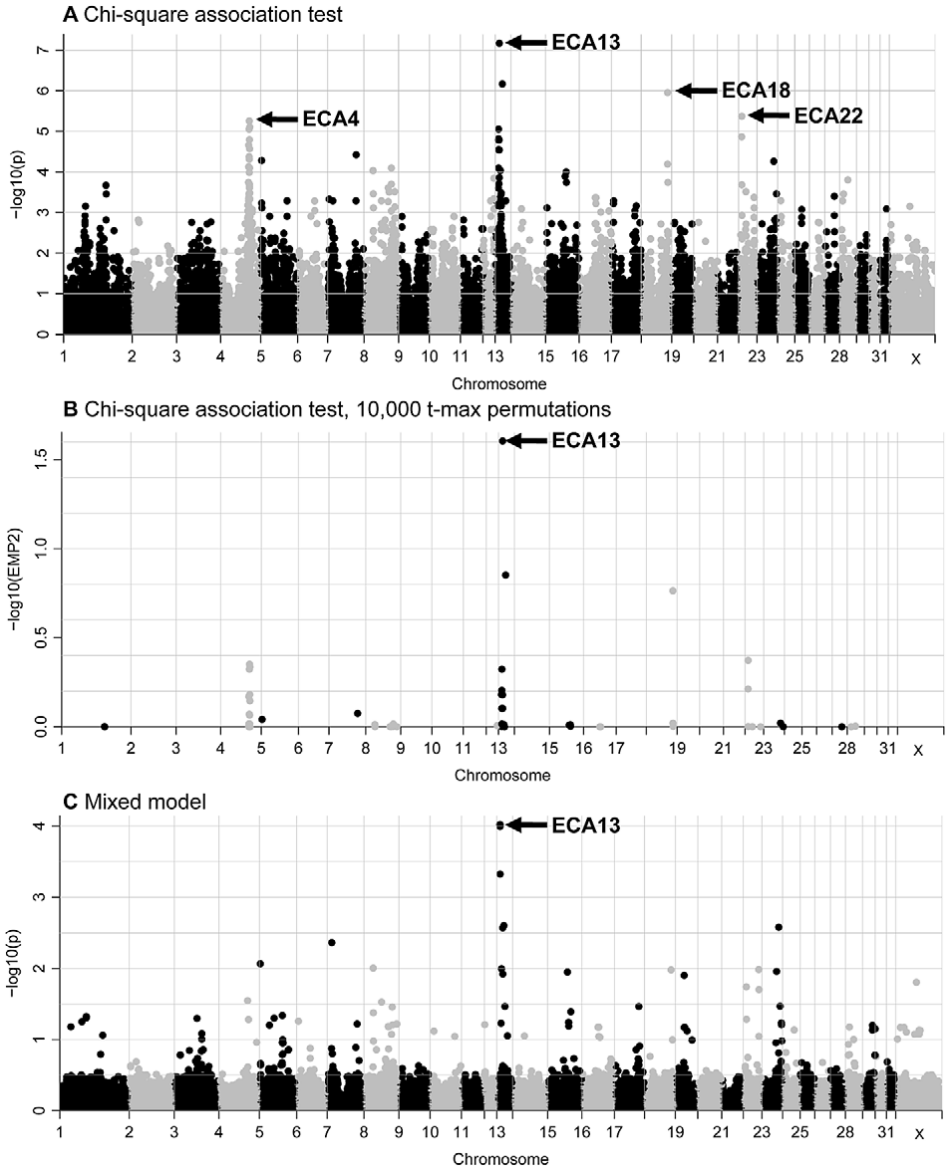
GWAS plots for the IAR phenotype displaying results for the 52,583 SNPs that passed quality control. (A) standard chi-square based association test without permutations; (B) standard association test after 10,000 t-max permutations, and (C) mixed-model test. The SNPs are plotted according to their position on each chromosome (x-axis) and association with the IAR phenotype (y-axis). Significance is given as the −log_10_ of the uncorrected (A and C) or corrected (B) *P*-value. The genome-wide significance threshold is log_10_ *P*>5 or log_10_ *EMP2*>1. 5.

The standard chi-square association test revealed high (*P* = 6.75E-08) to moderate (*P* = 9.26E-05) association with the IAR phenotype for 12 SNPs on ECA13 and moderate association (7.98E-05>*P*>4.23E-06) for 12 SNPs on ECA4 (Figure 1A). In addition, moderate associations with one or two SNPs were identified on five other chromosomes, ECA7, ECA15, ECA18, ECA22, and ECA23. The most significant 36 SNPs by the basic association test and their significance values by different regression analyses are presented in Table S2. After data correction by 10,000 t-max permutations, a single SNP on ECA13 was significant with a t-max permuted *P*<0.05 (Figure 1B). Because the small number of available cases precluded segregation analysis to determine the mode of inheritance, in addition to the chi-square allelic test of association, logistic regression models where marker genotypes were coded as additive, dominant or recessive were also tested. Association with ECA13 was evident in all three logistic regression tests (Figure S3), though the values remained below the significance threshold which was log_10_ *P*>5 or log_10_ *EMP2*>1. 5.

The genomic inflation factor (lambda = 1.31) in the chi-square analysis suggested that relatedness in the sample cohort was leading to inflation of the test statistic. Therefore, a mixed model analysis was conducted to account for relatedness and correct for inflation of the test statistic. Seven SNPs on ECA13 showed moderate evidence of association to IAR in the mixed model (1.0E-04>*P*>9.54E-05; Figure 1C and Table S2). No suggestive associations on other chromosomes were detected.

**Figure 2:**
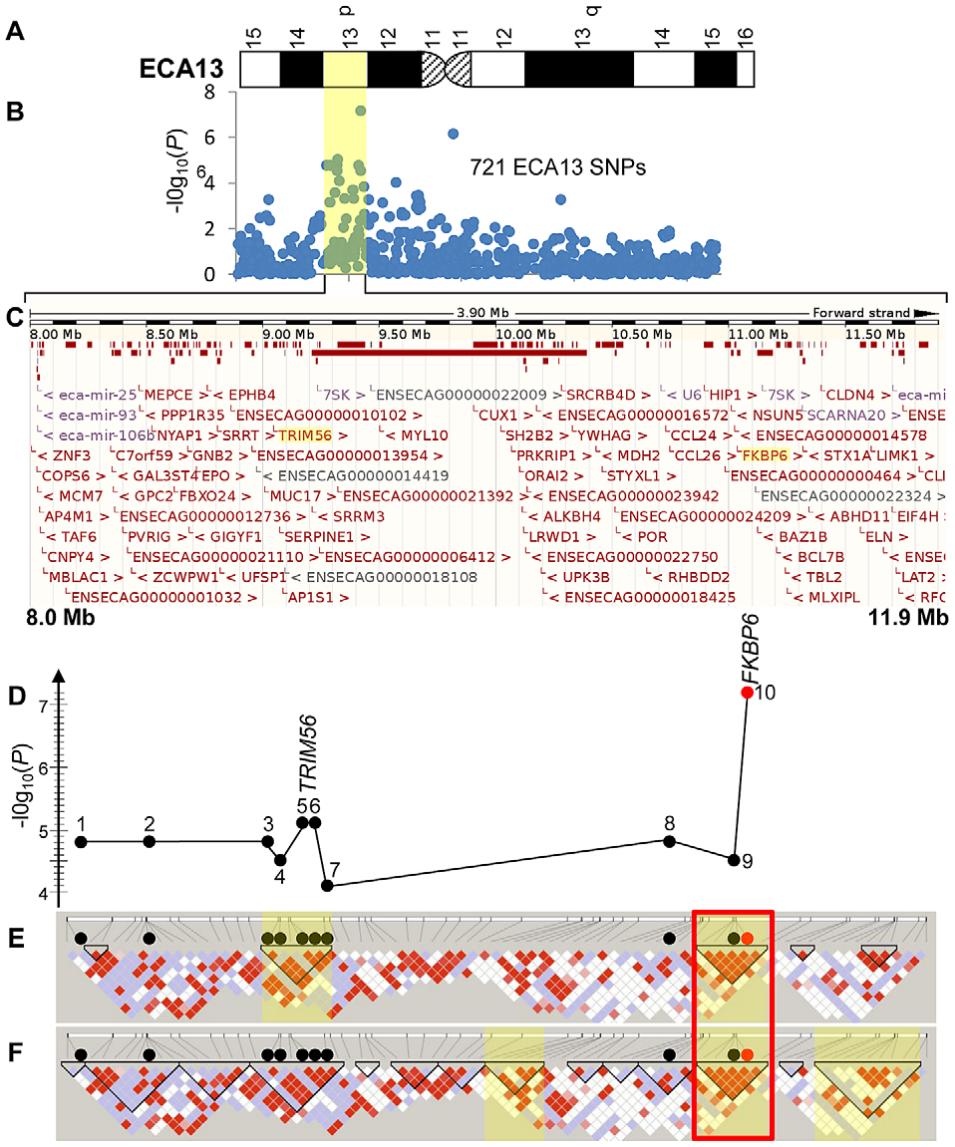
Schematic of the IAR–associated region in ECA13p. (A) A G-banded ideogram of ECA13 (ISCNH, 1997) showing the cytogenetic location of the IAR associated region; (B) Statistical significance values for 721 ECA13 SNPs analyzed for genome wide association using chi-square; yellow shade highlights the IAR-associated ∼3.9 Mb region; (C) Sequence map of chr13:8,000,000–11,900,000 showing all ENSMBL annotated genes in the region; *TRIM56* and *FKBP6* are highlighted. (D) A graph showing chi-square test −log_10_(*P*) values for 10 significant SNPs: 1–8027172, 2–8382955, 3–8977804, 4–8987922, 5–9034435, 6–9034502, 7–9183989, 8–10894213, 9–11043916 (black circles), and 10–11044175 (red circle); the seven SNPs above the grey line showed moderate significance also in mixed model; (E) Confidence interval LD blocks; (F) Solid spine LD blocks; blocks with significant permuted *P*-values (*P*<0.0005) for association with IAR are highlighted yellow; red diamonds represent D′ values equal to 1, lower values of D′ are presented in blue and white; the ∼306 kb highly associated haplotype in both LD analyses is in a red box.

In summary, association of the IAR phenotype to ECA13 was evident from all genome-wide studies. Next, statistical tests with the 721 ECA13 SNPs present on the SNP50 Beadchip showed that the most significant SNPs and their *P*-values varied between analyses (Figure S4 and Tables S2, S3). Yet, 7 SNPs (Figure 2; SNPs 1–3, 5, 6, 8, 10) had consistently strong to moderate association (4.7E-04>*P*>6.75E-08) with IAR by chi-square and mixed-model tests demarcating a 3.9 Mb association region in ECA13p.

### Haplotypes and linkage disequilibrium (LD)

Eightly-six (86) SNPs from Equine SNP50 Beadchip, representing an ECA13 region 8,023,293–11,902,171, were computationally phased and a single 3.31 Mb long haplotype from SNP 13:8,023,293 to SNP 13:11,334,980 was present in all IAR cases (Figure S5). Five affected stallions (HS03, HS29, HS30, HS33 and HS34) were homozygous while two stallions (HS31 and HS32) were heterozygous for the haplotype. The frequency of this extended haplotype was 0.86 in cases compared to 0.18 in controls.

Using the confidence interval (CI) method (Table S4), 5 haplotype blocks were defined in the region. Two of these blocks contained haplotypes that were strongly associated with IAR (Figure 2E shaded regions; Table S4). When haplotypes were defined using the solid spine (SS) of LD method, 14 haplotype blocks were identified of which three contained strongly associated haplotypes (Figure 2F shaded, Table S5). Notably, a ∼306 kb block at ECA13:11,028,316–11,334,980 bp with a single highly associated haplotype (*P* = 2.49E-08) was identical in the two LD analyses (Figure 2E, 2F; red box).

### Identification of *FKBP6* as a candidate gene

The 3.9 Mb highly associated region in ECA13 is extremely gene rich with 105 protein coding genes (27 genes per Mb), 4 RNA genes, and 6 pseudogenes (Figure 2B; Ensembl: http://www.ensembl.org/index.html). The region harbors over 2,126 SNPs in the Broad Institute database (http://www.broadinstitute.org/ftp/distribution/horse_snp_release/v2/) which makes re-genotyping unpractical. Therefore, for candidate gene discovery, we determined the closest genes in a 100 kb window around the best SNPs (Figure 2, Table S2) and the genes in haplotype blocks (Tables S6, S7) using BioMart (http://www.biomart.org/). Two genes, *FKBP6* and *TRIM56*, were selected for further analysis because intron 4–5 of *FKBP6* contained the only SNP (13:11,044,175) that retained significant association after permutations, and *TRIM56* was located ∼12 kb from moderately associated SNPs 13:9,034,435 and 13:9,034,502 (Figure 2). Functionally, *FKBP6* encodes a member of FK506 binding protein family and has been associated with male infertility in humans [32], [33], [34] and mice [33], while *TRIM56* belongs to tripartite motif-containing gene family, members of which, like *TRIM36* (*alias HAPRIN*), are involved in acrosome reaction of mammalian sperm [9], [17].

Individual PCR amplification and Sanger sequencing of the 3 exons of *TRIM56* and the 7 exons of *FKBP6* (Ensembl; http://www.ensembl.org/index.html) in all cases (n = 7) and selected Thoroughbred (TB) controls (n = 7) revealed two SNPs in *TRIM56*, and four SNPs in *FKBP6*, of which two were non-synonymous (Table 2). Polymorphisms in *TRIM56* were not significantly associated with IAR and the gene was rejected as a candidate locus, whereas association with IAR was found with *FKBP6* exon 4 and the gene was subjected for further analysis.

**Table 2:** Polymorphisms identified in the sequences of *FKBP6* and *TRIM56*.

Gene symbol	Discovery SNPs by GWAS	Polymorphisms (HGVS)*	Broad Institute database	Location in zthe gene	Substitution type
*FKBP6*	13:11044175	g.11038994A>G	new	Exon 3	synonymous
		g.11040315G>A	new	Exon 4	synonymous
		g.11040379C>A	*BIEC2-219719*	Exon 4	His>Asp; p.166H>N
		g.11047232T>C	*BIEC2-219729*	Exon 5	Cys>Arg; p.221C>R
*TRIM56*	13:9034435	g.9048610T>C	new	Exon 2	synonymous
	13:9034502	g.9048622G>A	new	Exon 2	synonymous

* Human Genome Variation Society nomenclature; http://www.hgvs.org/mutnomen/.

Case-control genotyping and association tests in the entire GWAS discovery cohort (n = 44) showed highly significant association of the IAR phenotype with *FKBP6* SNP g.11040315G>A (*P* = 1.51E-05), and significant association with SNP g.11040379C>A (p.166H>N; *P* = 0.004) (Table 3). This was further validated by sequencing exon 4 in a larger, multi-breed cohort (n = 265) of male horses with known (n = 94) and unknown (n = 171) reproductive phenotypes (Table S8). Homozygosity for the minor A-allele at g.11040315G>A was 100% in IAR affected stallions (n = 7), 0% in stallions with normal AR (n = 5), and only 9% in other fertile stallions (n = 82; Table S9). Likewise, all IAR stallions were homozygous for the major A-allele at g.11040379C>A; however this genotype was common in both stallions with normal AR (60%) and other fertile stallions (39%). While the IAR stallions were homozygous at both SNPs, this haplotype was rare (12%) across all horses tested (Table S9). The distribution of genotypes in the IAR cases and controls for the g.11040315G>A and g.11040379C>A alleles suggested a potential mode of inheritance for IAR, although this conclusion could not be unequivocally proven with this data. Therefore, tests of association between these alleles were computed using both genotypic and recessive coding. To test for association across g.11040315 and g.11040379, the combination of genotypes at both loci were used (Table 3). Fisher's exact tests were utilized due to the low number of counts in some cells of the contingency table. Overall, statistical analysis demonstrated highly significant associations with each SNP independently as well as the AAAA haplotype across both loci (Table 3; Table S9), whereas differences in allele frequencies among phenotypic groups were more pronounced for the synonymous SNP g.11040315G>A than for the non-synonymous change g.11040379C>A (p.166H>N). In summary, these findings suggested *FKBP6* as a susceptibility locus for IAR in stallions.

**Table 3:** Association analysis of *FKBP6* exon 4 and the IAR phenotype in three different study cohorts using Fisher's exact test over genotypic and recessive coding.

Study cohort	Model	*P*-values for g.11040315G>A	*P*-values for g.11040379C>A	*P*-values for both SNPs
GWAS cohort n = 44	genotypic	2.07E-05	0.00418383	4.13E-05
	recessive	2.07E-05	0.00131491	NA
Horses with known reproductive phenotypes n = 94	genotypic	3.35E-07	0.00908308	6.14E-08
	recessive	3.35E-07	0.00263567	NA
All horses n = 265	genotypic	3.20E-07	0.00365518	4.93E-11
	recessive	3.20E-07	0.00135393	NA

See also Tables S8 and S9 for detailed information about the study cohorts and association tests, respectively.

### Analysis of *FKBP6* expression

**Figure 3:**
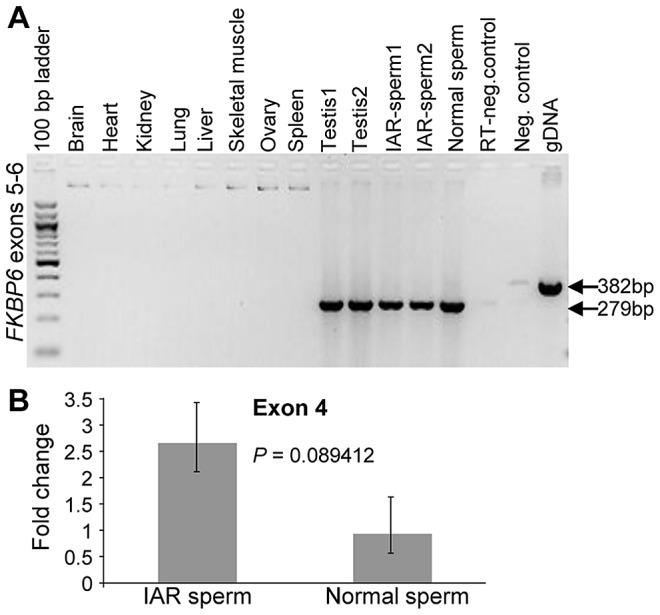
Expression analysis of equine *FKBP6*. (A) Reverse transcriptase (RT) PCR with *FKBP6* exons 5–6 spanning primers in a panel of 10 adult equine tissues; (B) Quantitative RT PCR showing moderate up-regulation of *FKBP6* in the sperm of IAR stallions.

Transcriptional profiling of equine *FKBP6* by RT-PCR in a panel of 10 adult equine body tissues revealed male limited expression in testis and sperm, including the sperm of IAR stallions (Figure 3A). Quantitative RT-PCR (qRT-PCR) with exon 4 primers showed low to moderate up-regulation of *FKBP6* in the sperm of IAR individuals compared to the sperm of normal stallions (Figure 3B), though the observed differences did not meet the 5% significance level (*P* = 0.08). Further studies with a larger number of samples are needed to determine whether there is a significant connection between the IAR phenotype and *FKBP6* up-regulation.

**Figure 4:**
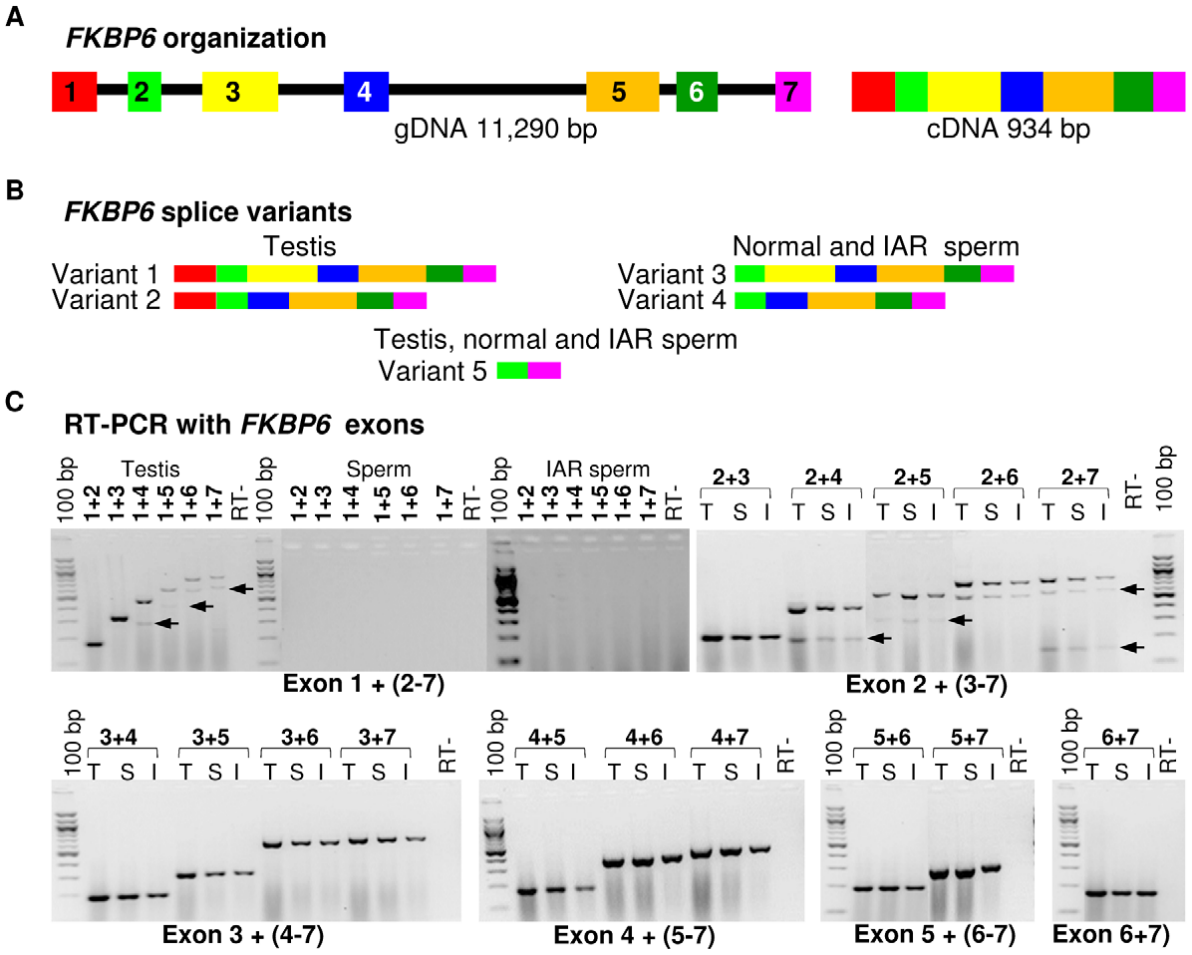
Equine *FKBP6* organization and splicing. (A) *FKBP6* genomic (left) and cDNA (right) according to ENSEMBL (http://www.ensembl.org/index.html); (B) *FKBP6* splice variants in stallion testis and sperm according to this study; (C) RT-PCR with combinations of *FKBP6* exon primers to determine alternative splicing in testis (T), normal sperm (S) and IAR sperm (I). Arrows show PCR products for alternative splice variants.

Next, we compared the expression of all seven *FKBP6* exons in testis, normal sperm and IAR sperm, and investigated the presence of splice variants. We observed that equine *FKBP6* has 5 splice variants – two in testis, two in sperm and one common for both (Figure 4; Figure S6). Of these, only one - the largest testis variant comprised of exons 1–7 - has been reported in Ensembl (http://www.ensembl.org/index.html), while transcripts omitting exon1, exon3, or comprised of exons 2 and 7 only, are novel findings and improve the current annotation of this equine gene. Notably, while minor differences in *FKBP6* transcripts (regarding exon 1) were seen between testis and sperm, no differences were observed between the transcripts in the sperm of normal and IAR stallions. We concluded that the sequence polymorphism that was strongly associated with the IAR phenotype shared no relationship with splicing.

**Figure 5:**
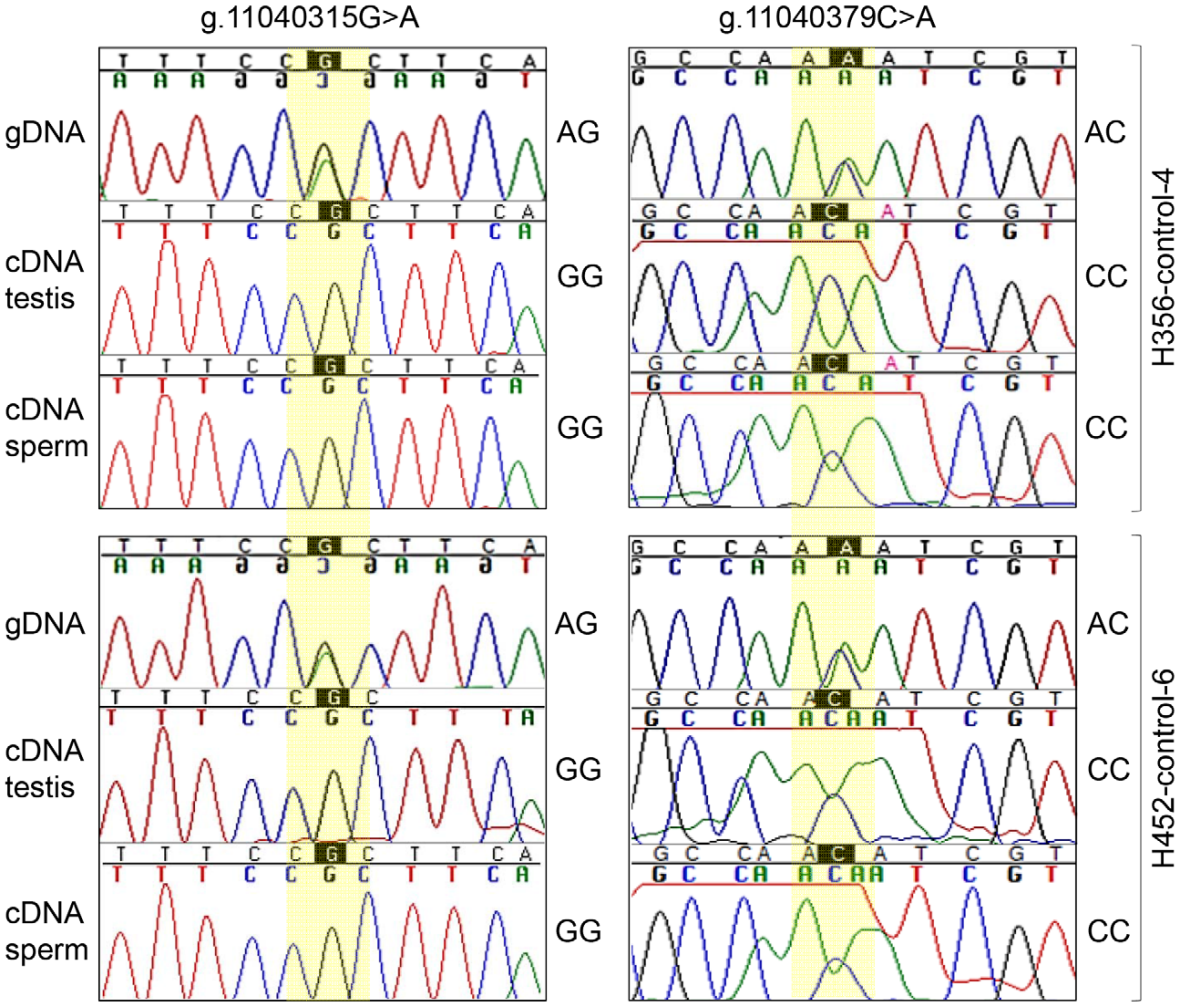
Monoallelic expression of equine *FKBP6*. gDNA and cDNA sequences from testis and sperm of two heterozygous control stallions.

Finally, we investigated possible allelic bias of *FKBP6* expression by direct cDNA sequencing from testis and sperm of one IAR stallion (HS03, the only one with available testis and sperm RNA) and control horses (n = 8) with heterozygous AGAC genotype for the exon 4 SNPs. As expected, cDNA of the IAR stallion was homozygous for the A-alleles. Remarkably, cDNA from heterozygous control animals showed only the G-allele at site g.11040315G>A, and the C-allele at site g.11040379C>A (Figure 5, Figure S7), suggesting monoallelic expression of the gene. However, because of the small sample size it is not clear at this point whether the expression of *FKBP6* is predominantly or exclusively monoallelic, and whether the allelic exclusion is random or always biased towards the non-A alleles. Due to the unavailability of parental genotypes, it also remains unclear whether *FKBP6* is a subject of genomic imprinting.

As an additional observation, the sequence reads of the cDNA, but not the gDNA, flanking g.11040379C>A consistently showed a ‘noisy’ pattern in all individuals regardless of the genotype (Figure 5, Figure S7) suggesting the presence of post-transcriptional mRNA/cDNA modifications at this site.

### *In silico* analysis of the equine FKBP6 protein

The *FKBP6* encodes for the FKBP6 protein (*alias* FKBP36) which is comprised of an N-terminal PPIase domain of the FKBP type encoded by exons 1–3, and a C-terminal domain containing three tetratrico peptide repeat (TPR) motifs, encoded by exons 4–7 (Ensembl: http://www.ensembl.org/index.html; Figure S8). Thus, SNP g.11040379C>A in exon 4, changing histidine to asparagine, is expected to affect the SMART SM00028 domain of TPR-repeat and Superfamily SSF48452 domain of TPR-like repeats (Figure S8). These domains are critical for trans-membrane protein-protein interactions and the formation of multiprotein complexes [35]. However, *in silico* analysis of the effect p.166H>N in exon 4 did not reveal any changes in the protein structure or the configuration of the trans-membrane segments.

## Discussion

Here we report about a successful application of the Equine SNP50 BeadChip [36] and GWAS for the discovery of a susceptibility gene for impaired acrosome reaction in stallions. The utility of this high density equine SNP genotyping platform has been previously validated for association mapping and phylogeny studies in horses and equids [36], for the discovery of a few single gene mutations [37], [38] and for the identification of genomic regions associated with complex traits and genetic disorders, such as osteochondrosis [39], [40], [41], neurological disorders [42], disease resistance [43], and athletic performance [44], [45]. However, to our best knowledge, this is the first application of the SNP50 genotyping platform for the discovery of genes associated with stallion subfertility. It is also among the very few GWAS for reproduction-related disorders in other mammals: previously, GWAS has been used for genetic analysis of impaired sperm functions in humans [46], [47] and pigs [48].

The key factor for detecting significant phenotype-genotype association in the small discovery population (n = 7) in this study was probably the affected horses being related Thoroughbreds. This horse breed originates from a small number of founders, has been a relatively closed population for the past 300 years [49], and is characterized by low genetic diversity, high inbreeding and the highest LD among horse breeds [36]. The latter facilitates GWAS which exploit pair-wise correlations between loci on a common genetic background not interrupted by recombination events [50]. Therefore, a much smaller number of genetic markers (or individuals) allowed effective capturing of information on the variants which were not genotyped. The downside of long LDs is that the associated regions are typically large making the discovery of causative genes/mutations difficult. Indeed, the associated region for IAR spanned about 3.9 Mb in ECA13p (Figure 2) and harbored over 100 genes and 2000 additional SNPs. The search for candidate genes was narrowed down to *FKBP6* because it was located in the region of most significant association with IAR both by GWAS and LD analyses (Figure 2). The involvement of *FKBP6* in male infertility in mice [33], [51] and humans [52] further strengthened the hypothesis.

Genomic organization, expression profile and domain structure of FK506-binding protein 6 (*FKBP6*) were first described in humans in connection with Williams syndrome [53]. The gene belongs to the immunophilins FKBP family of which all members feature two domains: an N-terminal prolyl isomerase/FK506-binding domain (a PPIase domain of the FKBP type) and a C-terminal protein-protein interaction domain containing three tetratrico peptide repeat (TPR) motifs [53], [54], [55]. The FKBP6 (*alias* FKBP36) protein is a component of synaptonemal complex and plays a critical role in male meiosis due to which *FKBP6* knockout male mice and rats are sterile with azoospermia, whereas females are reproductively normal [33], [51]. The role of *FKBP6* in human male fertility is more enigmatic. There are reports showing that *FKBP6* mutations do not cause azoospermia [32], [55], and those suggesting that *FKBP6* single nucleotide transitions play a modifying role in the susceptibility to idiopathic spermatogenic impairment [52]. At the same time, the expression of *FKBP6* in humans [32], rodents [33] and horses (this study) is limited to males and occurs predominantly or exclusively in testis – an indication of the involvement in spermatogenesis (meiosis) and maybe in sperm functions. The latter is supported by this study where we showed for the first time the presence of *FKBP6* transcripts in ejaculated stallion sperm, suggesting that the functions of this gene reach beyond meiotic prophase. Indeed, FKBP6 is thought to mediate the assembly of multiprotein complexes associated with clathrin-coated vesicles [54], such as acrosome or the synaptic vesicles. Further, through the C-terminal TPR domains, FKBP6 acts like a co-chaperone forming complexes with Hsp72, Hsp90, and GAPDH; GAPDH, in turn, promotes membrane-membrane fusion processes [56], e.g., the acrosome reaction [2]. Since FKBP6 acts as GAPDH inhibitor [56], we theorize that *FKBP6* transcriptional up-regulation, a tendency observed in the IAR stallions in this study (Figure 3), will negatively affect membrane fusion and acrosome reaction.

The FK506 binding proteins interact through protein-protein complexes with extracellular calcium-sensing receptors, such as ryanodine receptors (RyR), and are involved in maintenance of intracellular Ca^2+^ homeostasis and the control of membrane excitability in neuronal synapses [57]. If so, it is tempting to speculate that via interactions with calcium channels, FKBP6 could regulate acrosome exocytosis. In parallel to co-chaperone activity, complexes of FKBPs and Hsp90 facilitate interaction with steroid hormone receptors, thus potentially modifying the activity, stability and subcellular localization of the androgen receptor [34], [35], [53], [56].

Despite the evidence of the involvement of FK506 binding proteins in multiple biological processes, including spermatogenesis and fertilization, the current knowledge about FKBP6 is inadequate to properly interpret its role in sperm functions or understand the molecular link between *FKBP6* sequence polymorphism and IAR phenotype in this study. However, association of IAR with *FKBP6* exon 4 AAAA-genotype (Table 3; Table S9) and *FKBP6* monoallelic expression (Figure 5), inspire some ideas.

For example, it has been shown that post-translational protein modifications are encoded in the mRNA, so that even synonymous codons may provide subtly different information for the speed of translation and the nature of post-translational modifications [58], [59], [60]. It is therefore tempting to theorize that the SNPs g.11040315G>A and g.11040379C>A (p.166H>N) may affect transcription and the protein via chromatin-loops, transcription factor recruitment, or mRNA folding, whereas the A-allele might be a modifying factor. Special status of the A-allele is further emphasized by the monoallelic transcription of *FKBP6* and the preference for non-A-allele in heterozygotes (Figure 5, Figure S7). Our findings are consistent with the studies in humans where the expression of *FKBP6* is also allele-specific [32], and SNPs in the gene are thought to have a modifying effect on impaired spermatogenesis [52]. Despite this, causative relationship between the *FKBP6* and reproductive phenotypes in horses or humans need further understanding.

Subtle differences in the expression of two alleles have been detected in numerous non-imprinted human [61], [62], [63], [64] and murine [65] genes. In a few cases, allele-specific expression in low penetrance regulatory loci has been associated with genetic predisposition to disease [62]. Though the underlying mechanisms of allele-specific expression remain largely unknown, interaction of epigenetic factors with genomic variants in *cis* (within a gene) and *trans* (non-coding RNA genes, other regulatory elements) has been proposed. It is thought that some of the SNPs associated with disease phenotype by GWAS, might indicate the *trans*-regulators [62] giving another potential meaning to the *FKBP6* polymorphism.

Comparison of equine *FKBP6* DNA and protein sequences with human and other mammalian orthologs (http://www.ensembl.org/index.html) shows extensive sequence conservation (pairwise identity between horse and other species >92%; data not shown) but provides no clues to understand the genotype-phenotype association in this study: the polymorphic sites in horse exon 4 are monomorphic for the A-allele in human, mouse, cattle, pig and dog; also, *FKBP6* in other mammals comprises 8–9 exons compared to the 7 exons in horses.

Even though our findings clearly point at *FKBP6* as a susceptibility gene for IAR in stallions, its penetrance is incomplete, as revealed by genotyping and association analysis of 265 male horses from multiple breeds (Table 3, Tables S8, S9) showing that AA-genotype at the highly associated g.11040315G>A site was present in 9% of fertile stallions and 12% of other controls, and the AAAA-genotype was found in 2% of control stallions of known and unknown fertility. From another hand, incomplete penetrance, is in good agreement with the quantitative nature of IAR as demonstrated by Brinsko and colleagues (2007) showing that the failure of the AR in affected stallions is not complete and affects about 97% of the sperm, while the remaining 3% can perform normal AR. This could have led to phenotyping errors in the follow-up cohort where known fertility for stallions was quantified as at least one living offspring, whereas conception rates for each stallion were unavailable. Thus, it is conceivable that some of these stallions with the IAR susceptibility genotypes could have IAR, and only have living offspring as the result of multiple breeding attempts or assisted reproduction practices.

It is also plausible that either both sequence variants play a role in penetrance of the trait, or perhaps more likely, that the genotype is tagging an underlying functional variant. The results showing that the non-synonymous SNP (p.166H>N) causes no apparent change in protein structure and is less significantly associated with the IAR phenotype than the synonymous SNP, further support the possibility that *FKBP6* relates to the IAR phenotype via regulatory or modifying pathways. Besides, given the complexity of acrosome biogenesis and fertilization, it has been proposed that acrosome reaction is regulated by synergistic interactions of several sperm motility and sperm-egg adhesion genes [18] of which *FKBP6* might be one. This agrees with our initial uncorrected GWAS results showing moderate association of IAR to several horse chromosomes (Figure 1, Figure S3, Table S2). Among these, a ∼3.5 Mb region from 75 to 78.5 Mb on ECA4q might be of interest for future studies: though extremely gene poor, it contains *CADPS2* (Table S2) which encodes a SNARE-complex binding protein and is involved in vesicle exocytosis in the nervous system [66].

Male infertility/subfertility is a recognized concern in humans [67] and livestock species [68], whereas considerable proportion (∼25%) of unexplained male subfertility is caused by defects in sperm functions responsible for fertilization, including the acrosome reaction [20], [22], [24]. Implication of *FKBP6* as a susceptibility gene for IAR in stallions in this study, and evolutionary conservation of the gene across diverged mammalian species, suggest it as a new candidate gene for unexplained male subfertility for mammals, including humans. Importantly, because mouse and human fertilization are different [19] and human and horse sperm share more common features [1], molecular studies of fertility in stallions might provide valuable alternative to the rodent models in the search of molecular causes of infertility in man.

In summary, previous studies in humans [32], [47], [52] and the present results strongly suggest the involvement of *FKBP6* in various aspects of male fertility, molecular mechanisms of which remain yet to be fully explored. Nevertheless, testing prospective breeding stallions for *FKBP6* polymorphism is recommended to identify animals that might require specialized acrosome reaction analysis. This, in turn, may reveal more stallions with IAR, thus increasing the study cohort for future research. Breed-wise statistics of *FKBP6* genotype frequencies (Table 4) indicated that the likelihood finding the IAR susceptibility genotype AAAA is the highest in Thoroughbreds (7%) and the lowest in Quarter Horses (0%), while the 3% estimate for other breeds remains tentative because each of the 21 breeds studied was represented by only 1 to 8 individuals.

**Table 4:** *FKBP6* exon 4 genotype frequencies across horse breeds: Thoroughbred, TB (n = 145); Quarter Horse, QH (n = 56); other breeds (21 breeds; n = 64; 1 to 8 individuals each).

SNP	Genotype	Frequency		
		TB	QH	Other
**G>A**	AA	0.14	0.04	0.19
	AG	0.48	0.52	0.42
	GG	0.38	0.45	0.39
**C>A**	AA	0.39	0.45	0.36
	AC	0.48	0.39	0.34
	CC	0.13	0.16	0.30
**Both**	***AAAA***	***0.07***	***0.00***	***0.03***
	AAAC	0.06	0.02	0.06
	AACC	0.01	0.02	0.09
	AGAA	0.17	0.11	0.08
	AGAC	0.23	0.32	0.23
	AGCC	0.08	0.09	0.11
	GGAA	0.16	0.34	0.25
	GGAC	0.19	0.05	0.05
	GGCC	0.03	0.05	0.09

The IAR susceptibility genotype AAAA is in bold italics.

Last but not least, the study is one of the first examples of successful use of ejaculated sperm as the source of DNA and RNA for GWAS and gene expression analysis, thus propagating non-invasive sample procurement for fertility genomics in animals and humans.

## Materials and Methods

### Ethics statement

Procurement of stallion semen, peripheral blood and equine tissues was performed according to the *United States Government Principles for the Utilization and Care of Vertebrate Animals Used in Testing*, *Research and Training* and were approved by the Clinical Research Review Committee (CRRCs #08-19; #08-33; #09-32; #09-47) and Animal Use Protocol #2009-115 at Texas A&M University.

### Horses and phenotypes

The study cohort for genome wide association comprised of 44 male Thoroughbreds (breed purity confirmed by studbook data over 5 generations) - 7 cases and 37 normal male controls. The cases were initially identified due to unexplained subfertility (0–15% per cycle pregnancy rate) and diagnosed with IAR by incubating sperm with the calcium ionophore A23187 [25]. Acrosome reaction rate of the sperm in cases was 2.9% compared to 96% of sperm in normal stallions. Other sperm parameters, such as percentages of motile sperm and progressively motile sperm, as well as sperm morphologic characteristics, including percentages of abnormal heads and abnormal acrosomes, were in the normal range in the IAR stallions [25]. The control male horses (n = 37) were chosen from our Thoroughbred samples repository (n>200) so that they were not closely related (within a single generation) to the cases.

Follow-up genotyping for discovered variants in exons 4 and 5 of *FKBP6* was performed in a cohort of 263 horses from 20 known breeds or of mixed or unknown breed origin and 2 domestic donkeys. This cohort included 82 fertile male horses (including 37 controls for GWAS) that were known to be fertile defined as at least one viable offspring, 5 horses with known normal acrosomal reactions from Brinsko et al. 2007, 7 stallions with IAR, and 171 normal male individuals with unknown fertility status (Table S8). The majority of these horses were Thoroughbreds (n = 145) or Quarter Horses (n = 56) and originated from cytogenetic service lab DNA repositories (n>1000). Samples were coded numerically for confidentiality.

### Samples, DNA and RNA extraction

Snap frozen sperm pellets [25] were the only samples available for the IAR stallions, except stallion HS03 (Table 1), from whom testis and somatic tissues were procured by necropsy because the animal was euthanized due to health reasons. Control samples were selected from our equine DNA repository and were originally isolated from peripheral blood using standard protocols [69]. Samples for gene expression analysis came from our established equine tissue panels [70], and from our sperm/testis collections procured from reproductively normal stallions [71]. All tissue samples were stored in RNAlater (Ambion) at −80°C until use. Stallion sperm DNA was extracted as described by with the difference that the sperm were pre-processed with DTT solution (final conc. 8 µM) at 56°C for 1 h. Total RNA was extracted from sperm according to our detailed protocol [71] and from tissues using RNeasy Minikit (Qiagen). Sperm RNA was linearly amplified using using RampUp RNA Amplification Kit (Genisphere).

### Pedigree analysis

Data for 5-generation pedigrees of the 7 affected and 37 control Thoroughbreds was retrieved from *Pedigree Online Thoroughbred Database* (https://www.pedigreeonline.com/ped/renew.php). Multiple Trait Derivative Free Restricted Maximum Likelihood software, MTDFREML [72] was used to construct a pedigree and calculate inbreeding and genetic relationship coefficients among the seven affected (IAR) stallions using methods developed by Wright [73]. A 5-generation pedigree was drawn and pedigree-based statistics calculated in PEDIGRAPH software [74] for the 44 Thoroughbreds used for GWAS. Pedigree-based kinship and inbreeding coefficients were used to construct a relationship matrix for mixed model analyses (below).

### SNP genotyping, quality assurance, and detection of population stratification

The SNP genotyping and GWAS encompassed 44 male Thoroughbreds – 7 cases and 37 controls. The Equine SNP50 Beadchip [36] assay was performed at Neogen-GeneSeek, according to the manufacturer's standard procedures using 1 µg of genomic DNA (gDNA) per sample. The equine SNP chip comprised of 54,602 SNPs distributed on the 31 equine autosomes and X chromosome with a 43.2 kb average distance between markers.

Across the 44 samples the mean genotyping call rate was 97.8% (range 96.3% to 98.1%) and no animals were excluded from analysis. Genotypes were filtered using the PLINK toolset v1.06 [75]. A total of 41,521 SNPs passed quality control after excluding those with genotyping rates below 90% (n = 1922), deviating from Hardy-Weinberg equilibrium in controls (P<0.001; n = 154), with minor allele frequency<0.05 (n = 11,224), or having a differential case/control missingness (P<0.01; n = 38); 257 SNPs occurred in more than one category; 41,521 SNPs were used in all subsequent analyses.

In addition to pedigree analysis, the PLINK toolset was used to quantify population stratification based on pair-wise identity-by-state (IBS) distances from SNP genotypes [75]. Genetic distance (D) was calculated as D = 1−[(IBS2+0.5*IBS1)/N], where IBS2 and IBS1 are the number of loci that share either 2 or 1 alleles identical by state (IBS), respectively, and N is the number of loci. Metric multidimensional scaling (MDS) analysis of pair-wise genetic distances (6 dimensions) was used to identify the relationships between animals with PLINK (–mds-plot 6). Differences in IBS pair-wise distances within and between case and control cohorts were evaluated and empirical *P*-values were computed after 10,000 case/control label swapping permutations in PLINK. Co-ancestry coefficients were estimated from SNP data using PLINK by estimation of the proportion of the genome identical-by-decent (IBD) from P(IBD = 2)+0.5*P(IBD = 1), where P(IBD = 1) and P(IBD = 2) are the probabilities 1 or 2 alleles being IBD. SNP genotyping data are available at www.animalgenome.org/repository/pub/UMN2012.1105/.

### Association analyses

Unstructured genome-wide association tests were performed in PLINK using a basic case-control chi-square test as well as logistic modeling for additive, dominant, and recessive allelic effects. For all analyses, genomic inflation factor (GIF, lambda) was calculated and quantile-quantile plots (QQ plots) were generated to detect inflation of test statistics due to population stratification. Basic association and logistic regression analyses in PLINK were followed by 10,000 t-max permutations to correct for multiple testing.

To control for population structure and relatedness within the sample cohort, known pedigree relationships were accounted for using a logistic mixed model [76], which included a relationship matrix using inbreeding and kinship coefficients constructed from available 5 generation pedigree information using Pedigreemm (http://cran.r-project.org/web/packages/pedigreemm/pedigreemm.pdf) and SNPMatrix (http://bioconductor.org/packages/2.6/bioc/manuals/snpMatrix/man/snpMatrix.pdf) in the R environment (R Development Core Team, 2009). For GWAS, uncorrected *P*-values<5×10^−7^ and permutation *P*-values≤0.05 were considered as strong evidence of association [77]. Plots of GWAS results were generated in R (Figure 1, Figure S3).

### Haplotype analysis

Eighty six (86) SNPs from the region of interest were computationally phased using fastPHASE [78]. The extent of conserved haplotypes within cases was determined by visual inspection (Figure S5). Haplotype blocks across the entire cohort (cases and controls) were defined in Haploview v 4.1 [79] using both the confidence interval and solid spine of linkage disequilibrium (LD) methods [80]. For the confidence interval method, blocks were created when 90% of informative comparisons were in strong LD (i.e., had confidence interval minimum between 0.7 and 0.98), markers with MAF<0.01 were excluded; D′>0.8 was required to extend haplotype blocks using the strong spine of LD. For haplotype blocks called by both methods, haplotypic associations were computed using the chi-square statistic in Haploview. Correction for multiple testing was achieved by using 10,000 label swapping permutations within haplotypes in blocks to generate permuted *P*-values. The BioMart software suite (www.biomart.org) was used to query Ensembl, to identify genes within the haplotype boundaries from the *Equus caballus* (EquCab2) genome assembly.

### Candidate gene sequencing, mutation discovery, and association estimates

Equine genes from the significantly associated region and their human orthologs were identified and analyzed for functional relevance using the Ensemble (http://www.ensembl.org/index.html) and the UCSC genome browsers (http://genome.ucsc.edu/). Two candidate genes, *TRIM56* and *FKBP6* were selected for sequencing. Primers to amplify all exons and exon-intron boundaries were designed using horse WG sequence assembly *EquCab2* (http://www.ensembl.org/Equus_caballus/index.html) and Primer 3 software (http://frodo.wi.mit.edu/primer3/input.htm), and are listed in Table S10. The initial PCR reactions were carried out on the gDNA of cases (n = 7) and controls; the products were sequenced using BigDye (Applied Biosystems) chemistry and the reactions were resolved on an ABI-3730 capillary sequencer (Applied Biosystems). Sequences were analyzed for mutations in Sequencher V 4.7 software (GeneCodes Co).

Genotypic and recessive chi-square and Fisher's exact tests of association were performed in discovered SNPs suing the GWAS cohort to identify SNPs associated with IAR phenotype. Two statistically significant SNPs in *FKBP6* exon 4 were selected for further genotyping, first in the entire GWAS cohort (n = 44) and next, in a large cohort of 265 samples (Table S8). Genotypes were determined by direct Sanger sequencing of PCR products. Additionally, SNaPShot primers were designed (Table S10) and tested for efficiency on selected samples. Association between phenotype and SNP genotype were tested using chi-square and Fisher's exact tests. Genotypes were coded as genotypic or recessive (0, 1) for the analysis. Genotypic tests were also performed using the combined 2-locus genotypes for both exon 4 SNPs (Table 3, Table S9).

### *FKBP6* expression analysis

Primers for reverse transcriptase (RT) PCR were designed for all *FKBP6* exons (Table S10). In order to determine *FKBP6* expression profile, RT-PCR reactions were carried out with primers for exon 4 and intron spanning primers for exons 5 and 6 on RNA isolated from 10 equine tissues, *viz.*, brain, heart, kidney, lung, liver, skeletal muscle, ovary, spleen and testis. Comparison of *FKBP6* expression between cases and controls was carried out by quantitative real time (qRT) PCR with primers for exons 4 (Table S10). The qRT-PCR assays were carried out on a LightCycler 480 (Roche Diagnostics), and the results were analyzed with LightCycler 480 Software v1.5. Next, multiple primer combinations (Table S11) were used for RT-PCR on sperm and testis of cases (n = 4) and controls (n = 2) to identify *FKBP6* splice variants. The quality of testis and sperm RNA was checked by RT-PCR with intron-spanning primers for sperm- and testis-expressed genes *SPA17* (our unpublished data) and *PRM2* (Figure S9), and with a sperm-negative gene *PTPRC* [71]. Amplifications were performed in 15 µL reactions using Superscript III One-Step RT-PCR System and Platinum Taq DNA polymerase (Invitrogen Corporation) as described previously [71]. Finally, RNA from the sperm and/or testis of control horses (n = 8) and one case (HS03) was subjected to direct cDNA sequencing to determine allelic expression of *FKBP6* (Table S12). The controls were (AGAC) heterozygous and the case (AAAA) homozygous for the two exon 4 SNPs. The cDNA was synthesized from sperm/testis RNA using SuperScript VILO cDNA synthesis kit (Invitrogen, Carlsbad, CA, USA). Synthesis of cDNA was performed in two-step qRT-PCR by incubating at 25°C for 10 min, 42°C for 60 min and 85°C for 5 min. Synthesized cDNA was purified by Qiagen MinElute PCR purification kit according to manufacturers' protocol, and used as template to amplify *FKBP6* exon 4 by PCR. The PCR products were sequenced by BigDye chemistry and the sequences were analyzed in Sequencher 4.7 (Gene Codes) software.

### FKBP6 protein analysis

The effect of DNA sequence polymorphism on *FKBP6* amino acid sequence and protein functions was studied using DAS (Dense Alignment Surface method) algorithm to identify clear start and end configurations of transmembrane segments in both wild and mutated *FKBP6* amino acid sequence (http://www.sbc.su.se/~miklos/DAS/maindas.html). Initially, subcellular localization sites of *FKBP6* amino acid sequences were identified by WoLF PSORT software (http://wolfpsort.org/). These protein predictions were based on both known sorting signal motifs and amino acid content. Finally, conserved domains were detected using BLASTp (http://blast.ncbi.nlm.nih.gov/Blast.cgi).

## Supporting Information

Figure S1 Pedigree analysis. Pedigree of the 44 Thoroughbred horses used for GWAS in this study. Rectangles indicate males, ovals indicate females, diamonds indicate the 37 controls and red highlights indicate the 7 affected stallions. The pedigree has been generated in PEDIGRAPH software.
(PDF)

Figure S2 Multidimensional scaling (MDS) plots. Metric MDS analysis of pair-wise genetic distance (as described in Materials and Methods) was used to identify relationships between the 44 horses: 7 cases and 37 controls.
(TIF)

Figure S3 Genome-wide association plots for impaired acrosome reaction using logistic regression models: (A) additive model, (B) dominant model, and (C) recessive model. The SNPs are plotted according to their position on each chromosome (x-axis) and association with IAR (y-axis). Significance is given as the −log_10_ of the uncorrected *P*-value. While the SNPs with the highest −log_10_ values remain below the accepted significance level (−log10 (*P*)>5 or −log_10_ (*MP2*)>1.3, SNPs on ECA13 (arrows) stand out from the rest of the genome supporting the results of basic chi-square based association test and mixed-model analysis (see text and Figure 1 for details).
(TIF)

Figure S4 Significance values for ECA13 SNPs. Negative log *P*-values (y axis) for the 721 ECA13 SNPs (bp position×axis) on the beadchip analyzed for genome wide association using chi-square (A), mixed model (B), additive (C), dominant (D) and recessive (E) logistic regression tests. Yellow shade highlights the ∼3.9 Mb region in ECA13p which is associated with IAR (see also Table S3).
(TIF)

Figure S5 Computational phasing of 86 Equine SNP50 Beadchip SNPs in ECA13 from 8023293 to 11902171 bp. A single long 3.31 Mb haplotype from marker chr13:8023293 to marker chr13:11334980 was present in all IAR cases. Five affected stallions (HS03, HS29, HS30, HS33 and HS34) were homozygous for the haplotype while two stallions (HS31 and HS32; arrows) were heterozygous for the haplotype. The frequency of this extended haplotype was 0.86 in cases compared to 0.18 in controls.
(TIF)

Figure S6 *FKBP6* expression. Gel images showing RT-PCR results with *FKBP6* exon-specific primers in testis and sperm of normal and IAR stallions.
(TIF)

Figure S7 Monoallelic expression of equine *FKBP6*. Genomic and cDNA sequences of exon 4 SNPs in testis and/or sperm of one homozygous IAR stallion (HS03) and eight heterozygous controls.
(TIF)

Figure S8 The organization of equine *FKBP6* gene and protein domains. (A) Schematic of *FKBP6* exons and corresponding protein domains. The TPR repeats encoded by exon 4 are highlighted blue; (B and C) *In silico* analysis of the effect of non-synonymous substitution at g.11040379C>A (p.166H>N) on protein domains - amino acid change from histidine to asparagine does not cause any changes in the protein.
(TIF)

Figure S9 Testis and sperm RNA quality check. Agarose gel images showing RT-PCR results with intron-spanning primers of sperm- and testis-expressed genes *PRM2* and *SPA17*, and a sperm-negative gene *PTPRC*.
(TIF)

Table S1 Identity by descent (IBD) case-control label swapping comparison.
(DOCX)

Table S2 Genomic regions associated with IAR in Thoroughbred stallions. The 36 best SNPs are sorted by *P*-value for standard chi-square based association test. SNPs that demarcate the 3.9 Mb associated region on ECA13p are shaded gray; ns – not significant (*P*>0.05); na - odds ratio could not be computed. Closest genes to each SNP in a 100 kb window were retrieved by BioMart (http://www.biomart.org/).
(DOCX)

Table S3 Association analyses results for 721 ECA13 SNPs; top 20 hits for each analysis method are highlighted in blue; *P-values could not be calculated; **model fit with marker genotype did not provide information after fitting the model with just the relationship matrix.
(XLSX)

Table S4 ECA13 haplotypes calculated by confidence interval of LD and association tests.
(DOCX)

Table S5 ECA13 haplotypes calculated by solid spine of LD and association tests.
(DOCX)

Table S6 Genes within haplotype blocks defined by confidence interval of LD. Alternative shading is used to easily distinguish between blocks.
(DOCX)

Table S7 Genes within blocks defined by solid spine of LD. **genes in bold span more than one haplotype block. Alternative shading is used to easily distinguish between blocks.
(DOCX)

Table S8 List of horses/equids used for large cohort genotyping of *FKBP6* SNPs g.11040315G>A and g.11040379C>A, and association analysis. IAR - impaired acrosome reaction; AR – acrosome reaction; Fertile – horses with confirmed fertility records; Unknown – male horses (stallions and geldings) with no records of fertility or subfertility.
(DOCX)

Table S9 Allele and genotype frequencies of *FKBP6* exon 4 SNPs in GWAS (n = 44) and large (n = 265) study cohorts.
(DOCX)

Table S10 Horse *FKBP6* primers for PCR amplification and sequencing of individual exons, and for genotyping and qRT-PCR of exon 4.
(DOCX)

Table S11 *FKBP6* exon primers for the discovery of splice variants: expected and observed PCR amplicons in testis and sperm.
(DOCX)

Table S12 Equine *FKBP6* exon 4 cDNA sequencing: monoallelic expression in testis and sperm.
(DOCX)
