# Supplementary figures and images for: Genome-Wide Association Study Implicates Testis-Sperm Specific FKBP6 as a Susceptibility Locus for Impaired Acrosome Reaction in Stallions
Source: PLoS Genet. 2012 Dec 20;8(12):e1003139. doi: 10.1371/journal.pgen.1003139 (PMC3527208; doi:10.1371/journal.pgen.1003139)

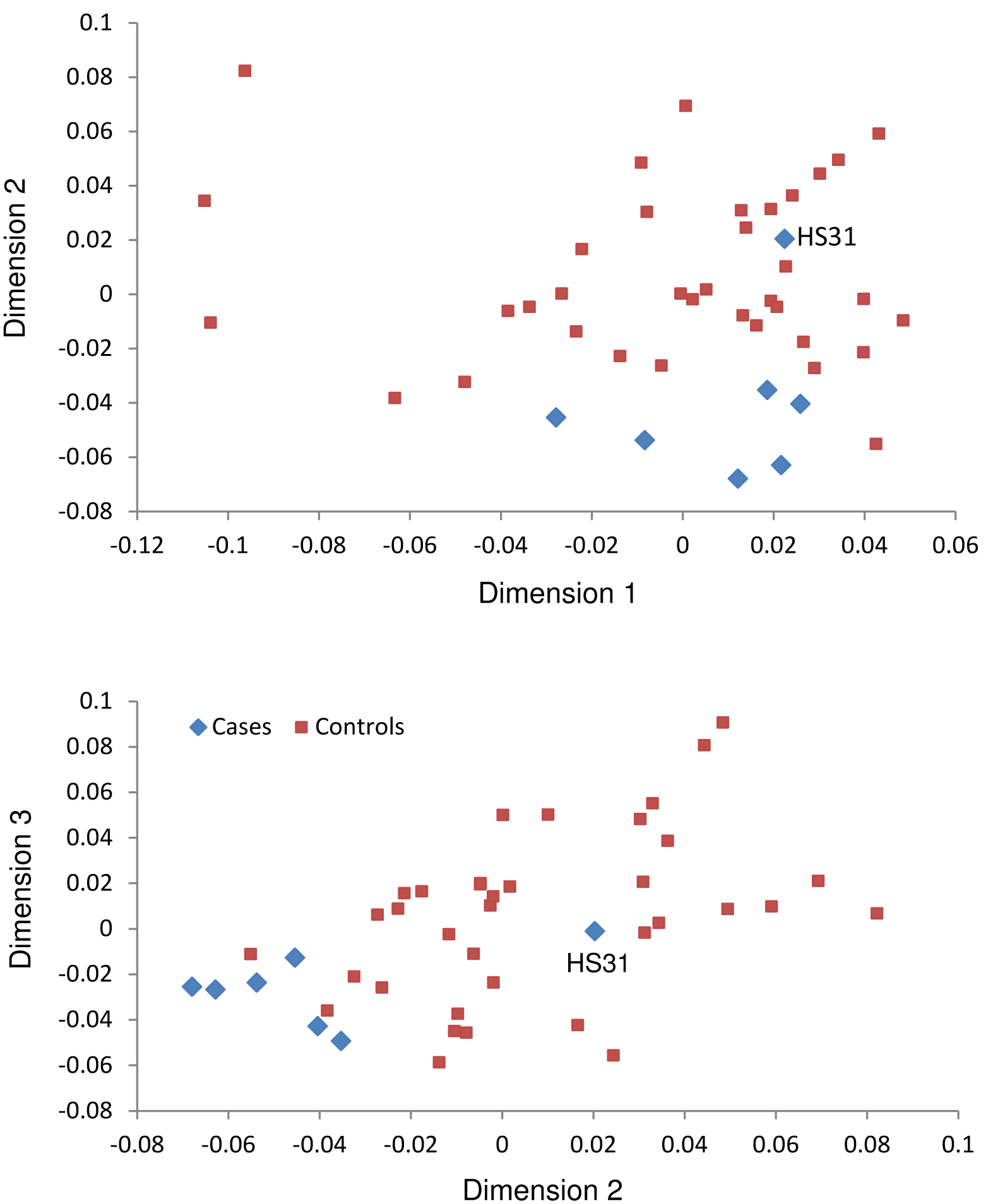

Supplement: Figure S2 — Multidimensional scaling (MDS) plots. Metric MDS analysis of pair-wise genetic distance (as described in Materials and Methods) was used to identify relationships between the 44 horses: 7 cases and 37 controls. (TIF) [file pgen.1003139.s002.tif]

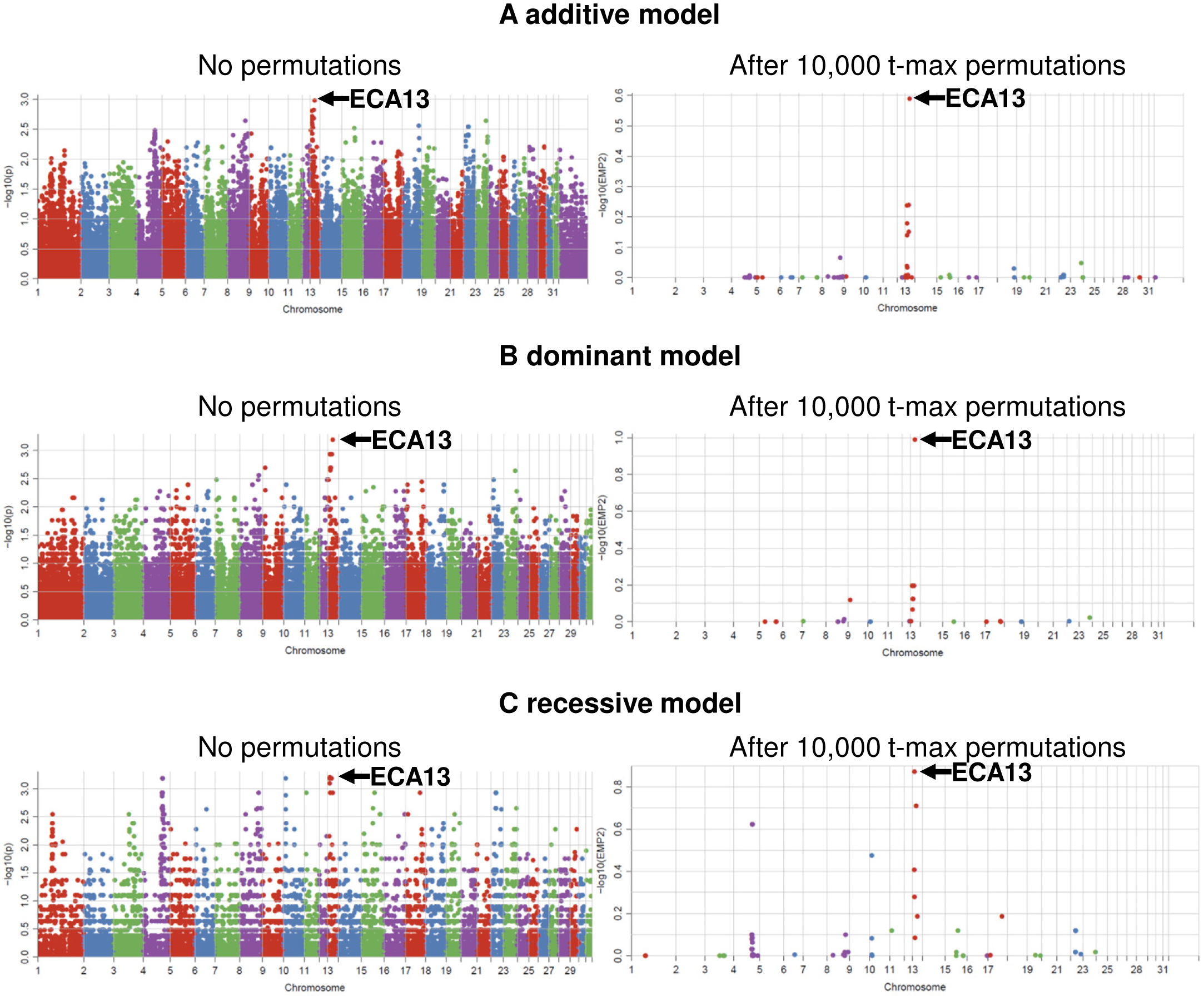

Supplement: Figure S3 — Genome-wide association plots for impaired acrosome reaction using logistic regression models: (A) additive model, (B) dominant model, and (C) recessive model. The SNPs are plotted according to their position on each chromosome (x-axis) and association with IAR (y-axis). Significance is given as the −log10 of the uncorrected P-value. While the SNPs with the highest −log10 values remain below the accepted significance level (−log10 (P)>5 or −log10 (MP2)>1.3, SNPs on ECA13 (arrows) stand out from the rest of the genome supporting the results of basic chi-square based association test and mixed-model analysis (see text and Figure 1 for details). (TIF) [file pgen.1003139.s003.tif]

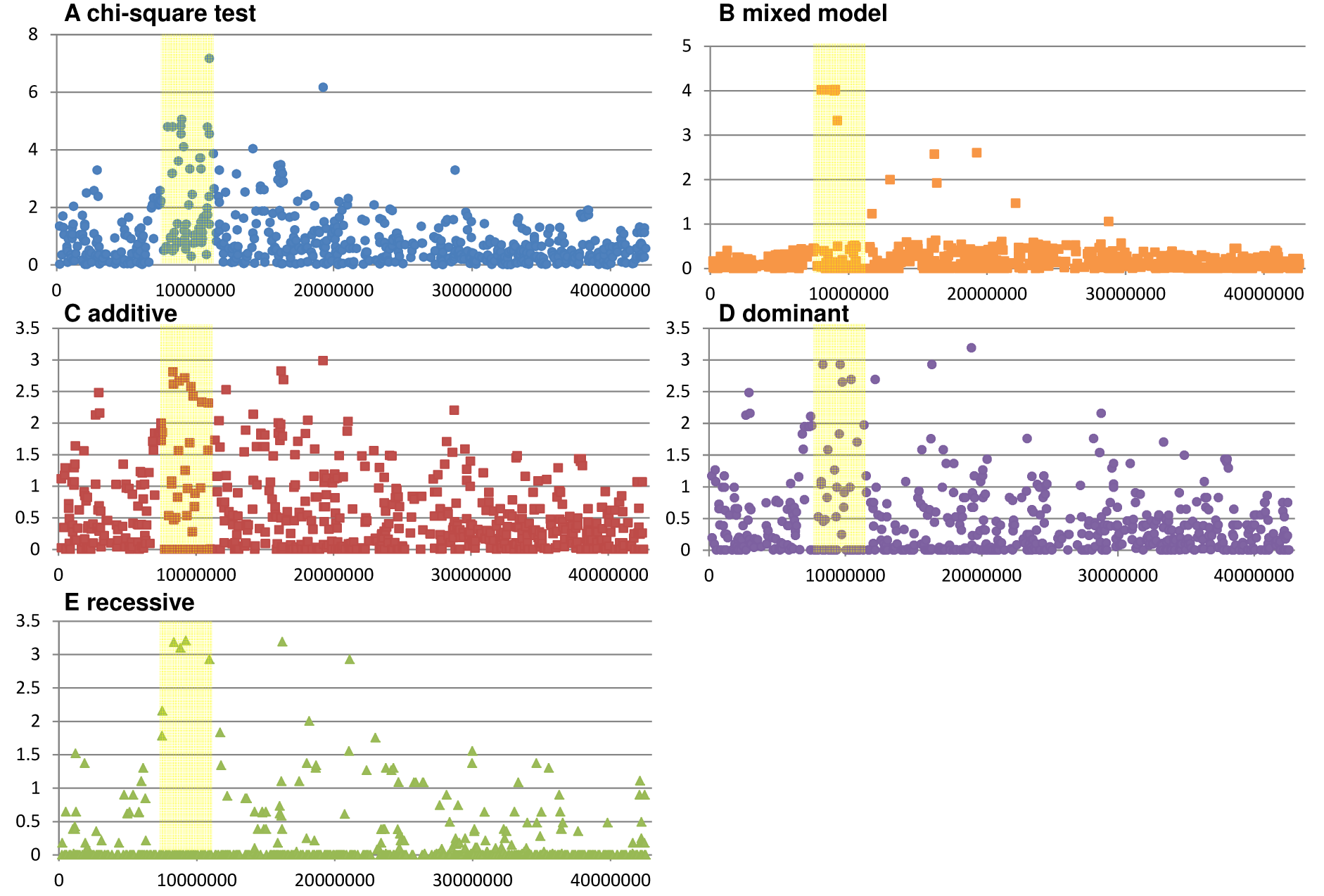

Supplement: Figure S4 — Significance values for ECA13 SNPs. Negative log P-values (y axis) for the 721 ECA13 SNPs (bp position×axis) on the beadchip analyzed for genome wide association using chi-square (A), mixed model (B), additive (C), dominant (D) and recessive (E) logistic regression tests. Yellow shade highlights the ∼3.9 Mb region in ECA13p which is associated with IAR (see also Table S3). (TIF) [file pgen.1003139.s004.tif]

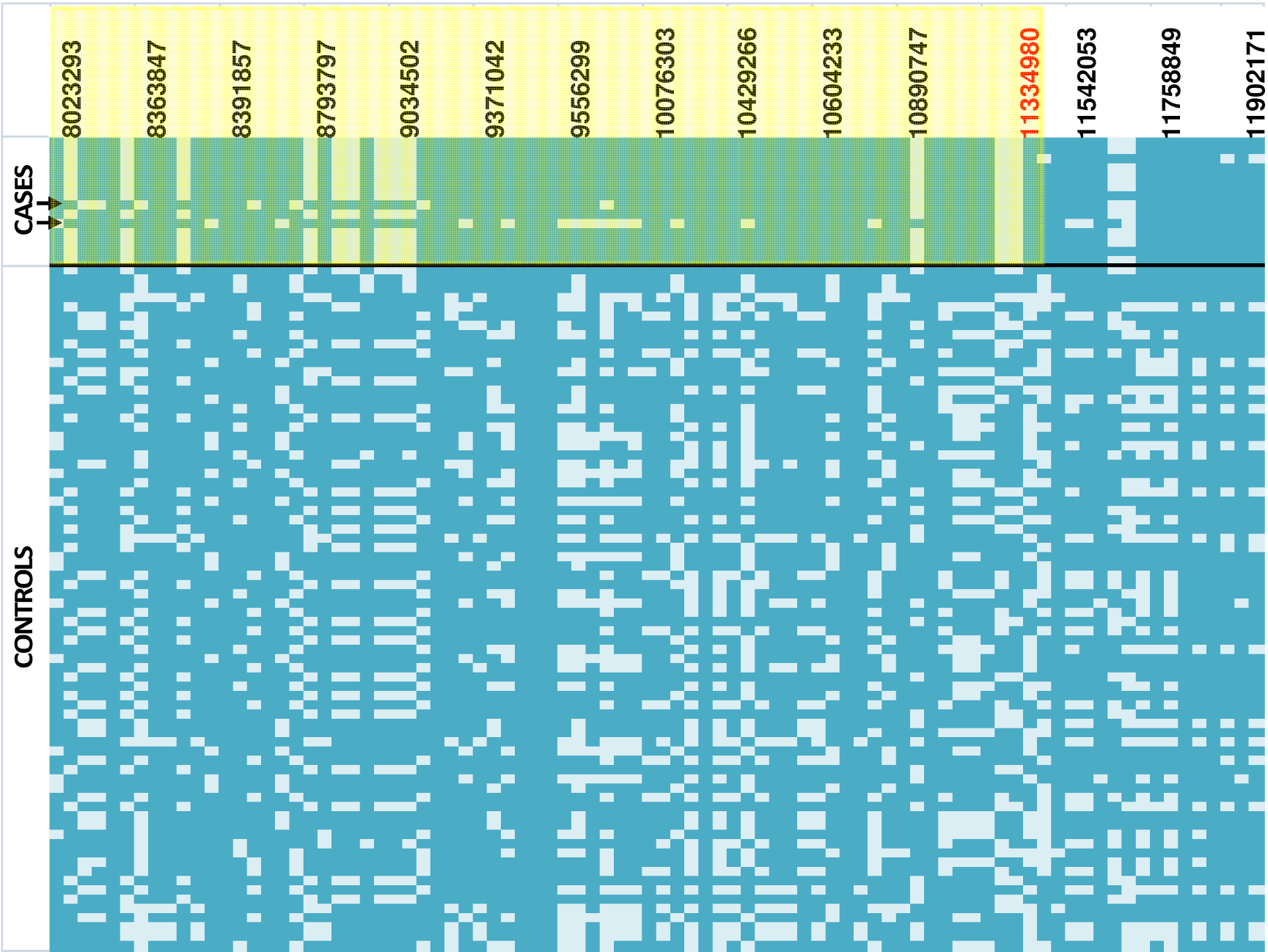

Supplement: Figure S5 — Computational phasing of 86 Equine SNP50 Beadchip SNPs in ECA13 from 8023293 to 11902171 bp. A single long 3.31 Mb haplotype from marker chr13:8023293 to marker chr13:11334980 was present in all IAR cases. Five affected stallions (HS03, HS29, HS30, HS33 and HS34) were homozygous for the haplotype while two stallions (HS31 and HS32; arrows) were heterozygous for the haplotype. The frequency of this extended haplotype was 0.86 in cases compared to 0.18 in controls. (TIF) [file pgen.1003139.s005.tif]

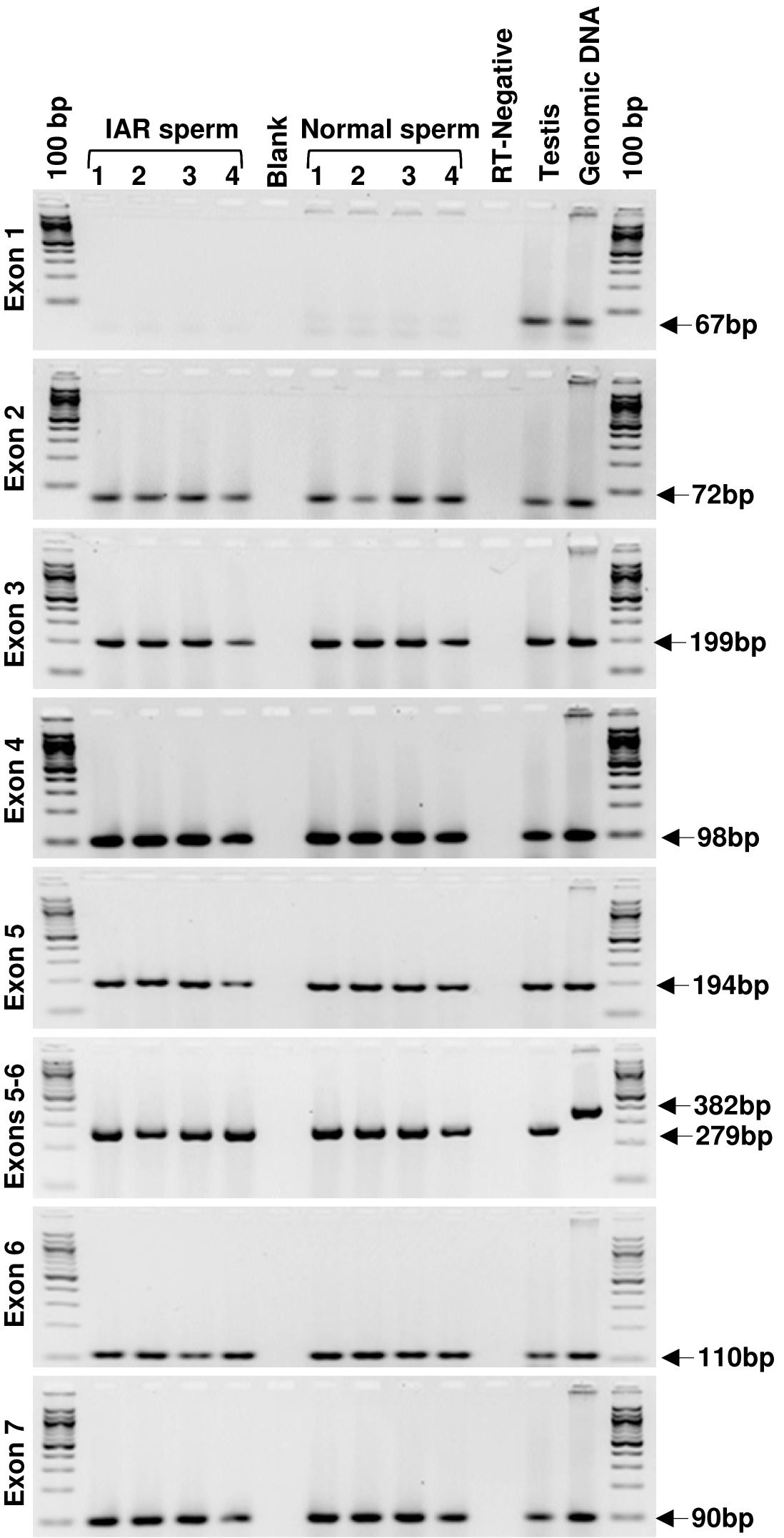

Supplement: Figure S6 — FKBP6 expression. Gel images showing RT-PCR results with FKBP6 exon-specific primers in testis and sperm of normal and IAR stallions. (TIF) [file pgen.1003139.s006.tif]

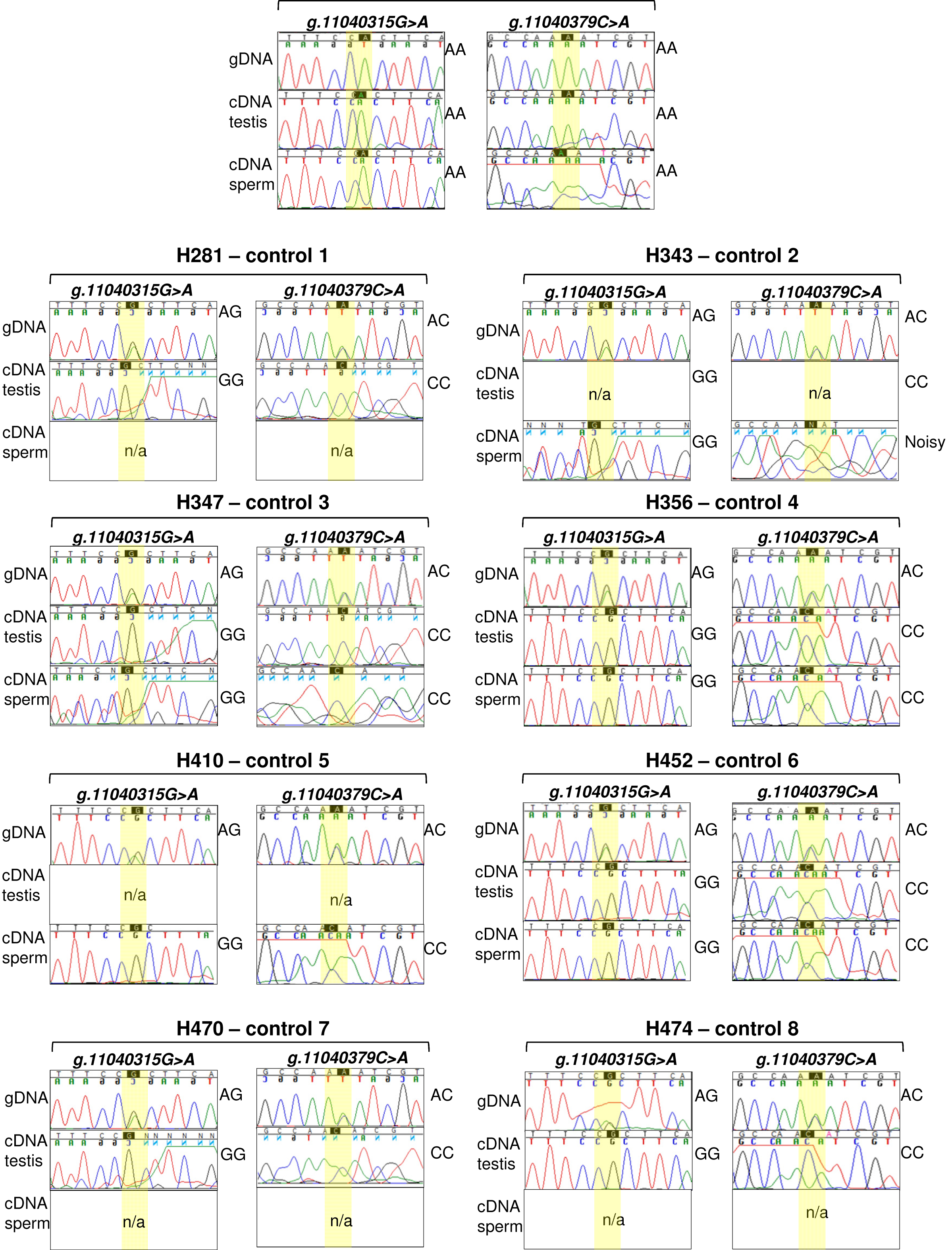

Supplement: Figure S7 — Monoallelic expression of equine FKBP6. Genomic and cDNA sequences of exon 4 SNPs in testis and/or sperm of one homozygous IAR stallion (HS03) and eight heterozygous controls. (TIF) [file pgen.1003139.s007.tif]

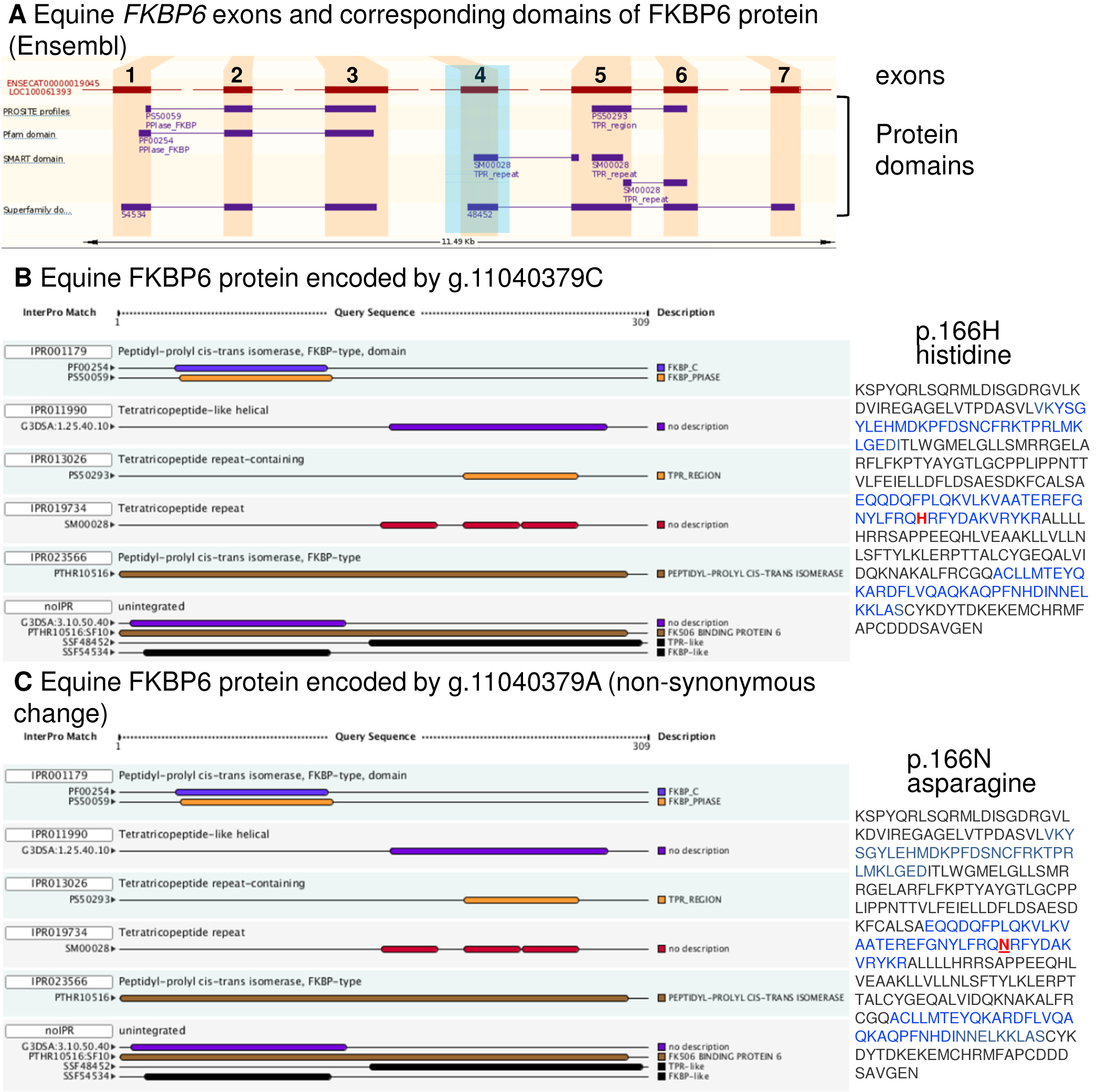

Supplement: Figure S8 — The organization of equine FKBP6 gene and protein domains. (A) Schematic of FKBP6 exons and corresponding protein domains. The TPR repeats encoded by exon 4 are highlighted blue; (B and C) In silico analysis of the effect of non-synonymous substitution at g.11040379C>A (p.166H>N) on protein domains - amino acid change from histidine to asparagine does not cause any changes in the protein. (TIF) [file pgen.1003139.s008.tif]

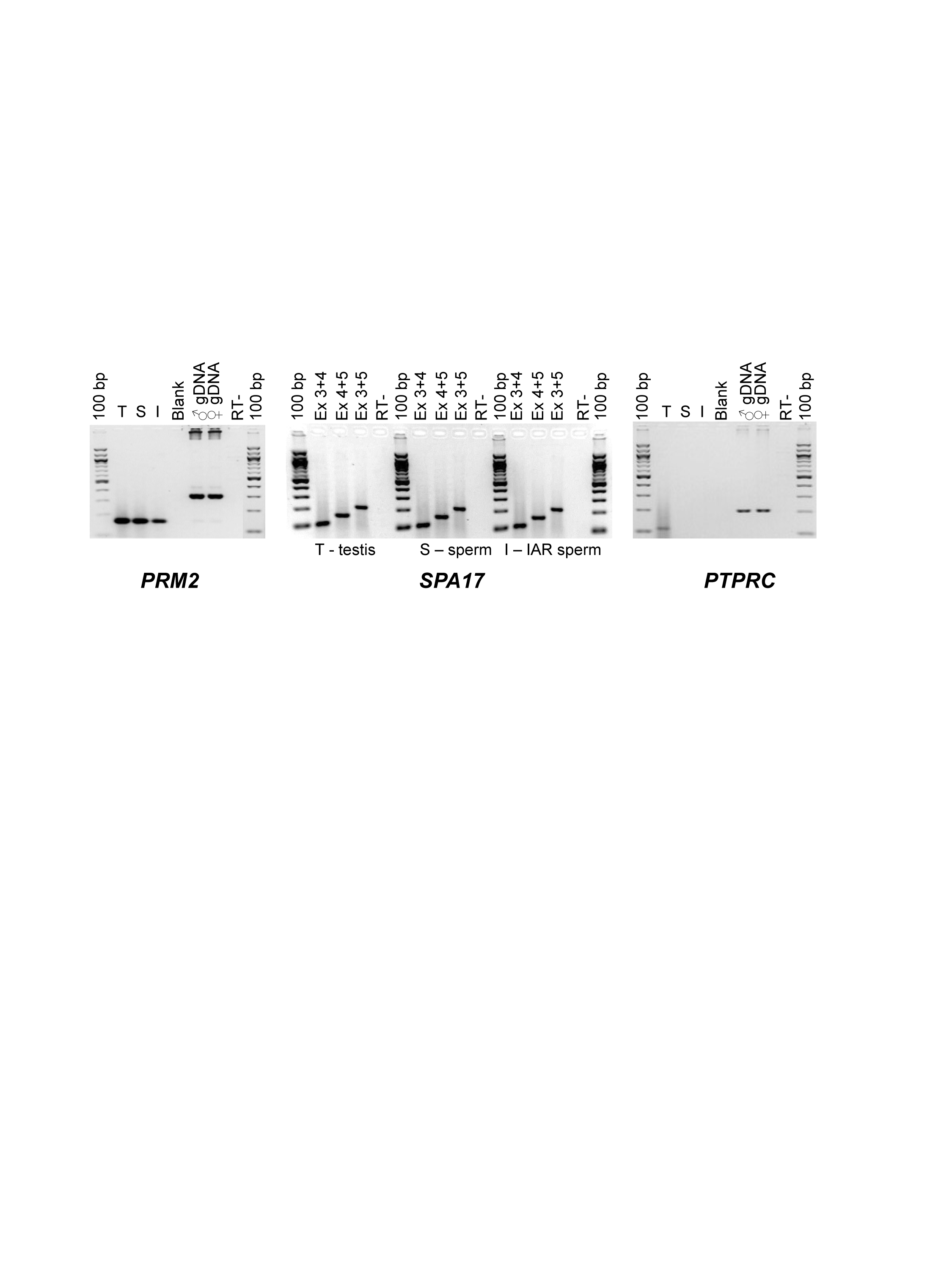

Supplement: Figure S9 — Testis and sperm RNA quality check. Agarose gel images showing RT-PCR results with intron-spanning primers of sperm- and testis-expressed genes PRM2 and SPA17, and a sperm-negative gene PTPRC. (TIF) [file pgen.1003139.s009.tif]
