# Supplementary material for: Genome-Wide Association Study Implicates Testis-Sperm Specific FKBP6 as a Susceptibility Locus for Impaired Acrosome Reaction in Stallions
Source: PLoS Genet. 2012 Dec 20;8(12):e1003139. doi: 10.1371/journal.pgen.1003139 (PMC3527208; doi:10.1371/journal.pgen.1003139)
Supplement: Table S1 — Identity by descent (IBD) case-control label swapping comparison. (DOCX) [file pgen.1003139.s010.docx]

**Table S1.** Identity by descent (IBD) case-control label swapping comparison

| **Comparison** | **p-value** |
| --- | --- |
| Case/control less similar | p = 0.931571 |
| Case/control more similar | p = 0.0684393 |
| Case/case less similar than control/control | p = 0.99904 |
| **Case/case more similar than control/control** | **p = 0.00096999** |
| Case/case less similar | p = 0.99997 |
| **Case/case more similar** | **p = 0.0000399** |
| **Control/control less similar** | **p = 0.00648994** |
| Control/control more similar | p = 0.99352 |
| Case/case less similar than case/control | p = 0.413096 |
| Case/case more similar than case/control | p = 0.586914 |
| Control/control less similar than case/control | p = 0.0208998 |
| Control/control more similar than case/control | p = 0.97911 |
