# Supplementary material for: Genome-Wide Association Study Implicates Testis-Sperm Specific FKBP6 as a Susceptibility Locus for Impaired Acrosome Reaction in Stallions
Source: PLoS Genet. 2012 Dec 20;8(12):e1003139. doi: 10.1371/journal.pgen.1003139 (PMC3527208; doi:10.1371/journal.pgen.1003139)
Supplement: Table S2 — Genomic regions associated with IAR in Thoroughbred stallions. The 36 best SNPs are sorted by P-value for standard chi-square based association test. SNPs that demarcate the 3.9 Mb associated region on ECA13p are shaded gray; ns – not significant (P>0.05); na - odds ratio could not be computed. Closest genes to each SNP in a 100 kb window were retrieved by BioMart (http://www.biomart.org/). (DOCX) [file pgen.1003139.s011.docx]

**Table S2.** **Genomic regions associated with IAR in Thoroughbred stallions.** The 36 best SNPs are sorted by *P*-value for standard chi-square based association test. SNPs that demarcate the 3 Mb associated region on ECA13p are shaded gray; ns – not significant (*P*>0.05); na - odds ratio could not be computed. Closest genes to each SNP in a 100 kb window were retrieved by BioMart (<http://www.biomart.org/>).

| **SNP**  **Chr:position (bp)** | ***P*-value chi-square** | **odds ratio chi-square** | **permuted *P*-value chi-square** | ***P*-value additive logistic regression** | ***P*-value dominant logistic regression** | ***P*-value recessive logistic regression** | ***P*-value mixed model** | **genes within 100 kb window** |
| --- | --- | --- | --- | --- | --- | --- | --- | --- |
| **chr13:**11044175 | 6.75E-08 | na | 0.0248 | ns | ns | ns | ns | *NSUN5 [ENSECAG00000012138], ENSECAG00000014578, FKBP6 [ENSECAG00000017859]* |
| **chr13:**19258278 | 6.79E-07 | 23.67 | 0.1404 | 0.00104 | 0.00065 | ns | 0.00249 |  |
| **chr18:**71300017 | 1.10E-06 | 18.4 | 0.1721 | 0.00278 | 0.00407 | ns | 0.01043 | *SLC39A10 [ENSECAG00000006518], DNAH7 [ENSECAG00000009699]* |
| **chr22:**8423735 | 4.23E-06 | 15.11 | 0.4228 | 0.00403 | 0.00330 | ns | ns | *ENSECAG00000000170* |
| **chr4:**77454283 | 5.53E-06 | 32.81 | 0.445 | 0.00326 | ns | 0.00065 | ns |  |
| **chr4:**78412704 | 7.83E-06 | na | 0.4604 | ns | ns | ns | ns | *FEZF1 [ENSECAG00000022989], CADPS2 [ENSECAG00000024021]* |
| **chr4:**77011798 | 8.84E-06 | 18.67 | 0.4744 | 0.00385 | ns | 0.00065 | 0.02828 |  |
| **chr13:**9034435 | 8.84E-06 | 18.67 | 0.4744 | ns | ns | ns | 9.54E-05 | *ENSECAG00000014539, TRIM56 [ENSECAG00000019517], F7DDC0_HORSE [ENSECAG00000019781]* |
| **chr13:**9034502 | 8.84E-06 | 18.67 | 0.4744 | ns | ns | ns | 9.54E-05 | *ENSECAG00000014539, TRIM56 [ENSECAG00000019517], F7DDC0_HORSE [ENSECAG00000019781]* |
| **chr22:**8075458 | 1.36E-05 | 12.76 | 0.6124 | 0.00502 | 0.00508 | ns | 0.01809 |  |
| **chr13:**8977804 | 1.52E-05 | 29.7 | 0.6237 | ns | ns | ns | 0.00010 | *MUC3A [ENSECAG00000011278], ENSECAG00000013954, ENSECAG00000014539* |
| **chr4:**77957097 | 1.61E-05 | 17.37 | 0.6583 | 0.00407 | ns | 0.00065 | ns |  |
| **chr13:**8027172 | 1.61E-05 | 17.37 | 0.6583 | ns | ns | ns | 9.58E-05 | *ZNF3 [ENSECAG00000024649], COPS6 [ENSECAG00000008997], F7C432_HORSE [ENSECAG00000015149], AP4M1 [ENSECAG00000016103], TAF6 [ENSECAG00000008522], CNPY4 [ENSECAG00000021787], MBLAC1 [ENSECAG00000006208]* |
| **chr13:**8382955 | 1.61E-05 | 17.37 | 0.6583 | ns | ns | ns | 9.58E-05 | *C7orf59 [ENSECAG00000006312], GAL3ST4 [ENSECAG00000004503], F6TL38_HORSE [ENSECAG00000008170], ENSECAG00000012736, PVRIG [ENSECAG00000020068], ENSECAG00000021110* |
| **chr13:**10894213 | 1.62E-05 | 28.83 | 0.6605 | 0.00486 | ns | 0.00118 | ns | *HIP1 [ENSECAG00000022880]* |
| **chr4:**75575124 | 2.17E-05 | 28.6 | 0.676 | 0.00563 | ns | 0.00135 | ns |  |
| **chr4:**78238792 | 2.67E-05 | 27.08 | 0.7141 | 0.00733 | ns | 0.00203 | ns | *AASS [ENSECAG00000021479]* |
| **chr13:**8987922 | 2.84E-05 | 16.2 | 0.7877 | ns | ns | ns | 9.60E-05 | *MUC3A [ENSECAG00000011278], ENSECAG00000013954, ENSECAG00000014539* |
| **chr13:**11043916 | 2.85E-05 | na | 0.7877 | ns | ns | ns | ns | *NSUN5 [ENSECAG00000012138], ENSECAG00000014578, FKBP6 [ENSECAG00000017859]* |
| **chr7:**76899332 | 3.76E-05 | 11 | 0.8407 | 0.00610 | 0.00749 | ns | ns | *OLFML1 [ENSECAG00000012937], PPFIBP2 [ENSECAG00000012953]* |
| **chr4:**76708998 | 4.21E-05 | 12.29 | 0.8493 | 0.00429 | 0.04325 | 0.00118 | ns |  |
| **chr4:**76971434 | 4.28E-05 | 25.48 | 0.853 | 0.00425 | ns | 0.00118 | ns |  |
| **chr4:**77084810 | 4.28E-05 | 25.48 | 0.853 | 0.00425 | ns | 0.00118 | ns | *ENSECAG00000001457, TSPAN12 [ENSECAG00000001545]* |
| **chr4:**77481610 | 4.83E-05 | 15.14 | 0.8603 | 0.00439 | ns | 0.00065 | ns | *[ENSECAG00000002740]* |
| **chr5:**2444291 | 5.20E-05 | 10.31 | 0.9087 | 0.00686 | 0.01983 | ns | 0.00858 | *C1orf186 [ENSECAG00000013660], F7BNU0_HORSE [ENSECAG00000013720], ENSECAG00000026830* |
| **chr5:**2449200 | 5.20E-05 | 10.31 | 0.9087 | 0.00686 | 0.01983 | ns | 0.00858 | *C1orf186 [ENSECAG00000013660], F7BNU0_HORSE [ENSECAG00000013720], ENSECAG00000026830* |
| **chr23:**45189440 | 5.47E-05 | 29.2 | 0.9529 | 0.00229 | 0.00229 | ns | 0.00262 | *MOB3B [ENSECAG00000021621]* |
| **chr18:**71454567 | 6.41E-05 | 12.69 | 0.9538 | 0.00697 | 0.03193 | 0.00414 | ns | *DNAH7 [ENSECAG00000009699]* |
| **chr4:**75637735 | 7.50E-05 | 11.41 | 0.9626 | 0.00479 | ns | 0.00118 | ns |  |
| **chr4:**77988671 | 7.98E-05 | 14.18 | 0.9639 | 0.00449 | ns | 0.00065 | ns |  |
| **chr8:**73994133 | 7.98E-05 | 14.18 | 0.9639 | 0.00449 | ns | 0.00224 | ns |  |
| **chr13:**9183989 | 7.98E-05 | 14.18 | 0.9639 | 0.00193 | ns | 0.00062 | 0.00047 | *PLOD3 [ENSECAG00000021913], ZNHIT1 [ENSECAG00000007913], CLDN15 [ENSECAG00000011386], FIS1 [ENSECAG00000013191], SRRM3 [ENSECAG00000022142], RABL5 [ENSECAG00000019190]* |
| **chr8:**24411688 | 9.26E-05 | 9.63 | 0.9705 | ns | ns | ns | ns |  |
| **chr8:**24528856 | 9.26E-05 | 9.63 | 0.9705 | ns | ns | ns | ns | *DHX37 [ENSECAG00000007404], BRI3BP [ENSECAG00000015008], AACS [ENSECAG00000015112]* |
| **chr13:**14182668 | 9.26E-05 | 9.63 | 0.9705 | 0.00729 | ns | ns | ns |  |
| **chr15:**56115725 | 9.93E-05 | 9.833 | 0.9731 | 0.00436 | ns | 0.01463 | ns | *EML4 [ENSECAG00000007410]* |
