# Supplementary material for: Genome-Wide Association Study Implicates Testis-Sperm Specific FKBP6 as a Susceptibility Locus for Impaired Acrosome Reaction in Stallions
Source: PLoS Genet. 2012 Dec 20;8(12):e1003139. doi: 10.1371/journal.pgen.1003139 (PMC3527208; doi:10.1371/journal.pgen.1003139)
Supplement: Table S4 — ECA13 haplotypes calculated by confidence interval of LD and association tests. (DOCX) [file pgen.1003139.s013.docx]

**Table S4.** ECA13 haplotypes calculated by confidence interval of LD and association tests

| **Block (number of SNPs)** | **from (bp)** | **to (bp)** | **size (kb)** | **haplotypes** | **haplotype frequency** | **case frequency** | **control frequency** | ***P* value (chi square)** | **permuted *P*-value** |
| --- | --- | --- | --- | --- | --- | --- | --- | --- | --- |
| Block 1 (2) | 8228067 | 8229250 | 1.183 | GC | 0.761 | 0.929 | 0.73 | 0.1095 | 0.802 |
|  |  |  |  | AT | 0.239 | 0.071 | 0.27 | 0.1095 | 0.802 |
| Block 2 (6) | 8977804 | 9183989 | 206.185 | **GCCGCG** | **0.602** | **0.143** | **0.688** | **1.00E-04** | **0.0074** |
|  |  |  |  | **AACATA** | **0.341** | **0.857** | **0.243** | **8.84E-06** | **0.0005** |
|  |  |  |  | GCTGCA | 0.035 | 0 | 0.041 | 0.4386 | 1 |
|  |  |  |  | AACGCG | 0.012 | 0 | 0.014 | 0.6521 | 1 |
|  |  |  |  | AATGCA | 0.011 | 0 | 0.013 | 0.6718 | 1 |
| Block 3 (6) | 11028316 | 11334980 | 306.664 | **TAATAT** | **0.349** | **1** | **0.226** | **2.49E-08** | **<0.0001** |
|  |  |  |  | CGCCGC | 0.205 | 0 | 0.243 | 0.0385 | 0.5613 |
|  |  |  |  | CGACGC | 0.125 | 0 | 0.149 | 0.123 | 0.8487 |
|  |  |  |  | TAATGT | 0.102 | 0 | 0.122 | 0.1684 | 0.9153 |
|  |  |  |  | TAACGC | 0.099 | 0 | 0.118 | 0.1764 | 0.9364 |
|  |  |  |  | TAACGT | 0.083 | 0 | 0.099 | 0.22 | 0.9612 |
|  |  |  |  | TAATGC | 0.023 | 0 | 0.027 | 0.5341 | 1 |
|  |  |  |  | TAATAC | 0.015 | 0 | 0.018 | 0.6178 | 1 |
| Block 4 (2) | 11542053 | 11557747 | 15.694 | CC | 0.739 | 0.929 | 0.703 | 0.0778 | 0.7862 |
|  |  |  |  | TT | 0.216 | 0.071 | 0.243 | 0.1519 | 0.8878 |
|  |  |  |  | TC | 0.045 | 0 | 0.054 | 0.3733 | 0.9962 |
| Block 5 (2) | 11758849 | 11770192 | 11.343 | GCG | 0.58 | 1 | 0.5 | 5.00E-04 | 0.0137 |
|  |  |  |  | GTG | 0.216 | 0 | 0.257 | 0.0323 | 0.5155 |
|  |  |  |  | ATA | 0.17 | 0 | 0.203 | 0.0644 | 0.7628 |
|  |  |  |  | GTA | 0.034 | 0 | 0.041 | 0.4433 | 1 |
