# Supplementary material for: Genome-Wide Association Study Implicates Testis-Sperm Specific FKBP6 as a Susceptibility Locus for Impaired Acrosome Reaction in Stallions
Source: PLoS Genet. 2012 Dec 20;8(12):e1003139. doi: 10.1371/journal.pgen.1003139 (PMC3527208; doi:10.1371/journal.pgen.1003139)
Supplement: Table S5 — ECA13 haplotypes calculated by solid spine of LD and association tests. (DOCX) [file pgen.1003139.s014.docx]

**Table S5:** ECA13 haplotypes calculated by solid spine of LD and association tests.

| **Block (number of SNPs)** | **from (bp)** | **to (bp)** | **size (kb)** | **haplotypes** | **haplotype frequency** | **case frequency** | **control frequency** | **p value (chi square)** | **permuted p-value** |
| --- | --- | --- | --- | --- | --- | --- | --- | --- | --- |
| Block 1 (2) | 8023293 | 8027172 | 3.88 | **CG** | **0.477** | **0.071** | **0.554** | **9.00E-04** | **0.0404** |
|  |  |  |  | **CA** | **0.352** | **0.857** | **0.257** | **1.61E-05** | **0.0011** |
|  |  |  |  | AG | 0.17 | 0.071 | 0.189 | 0.2826 | 0.9999 |
| Block 2 (8) | 8228067 | 8385924 | 157.86 | **GCAGGCGA** | **0.352** | **0.857** | **0.257** | **1.61E-05** | **0.0011** |
|  |  |  |  | ATGTGCAA | 0.216 | 0.071 | 0.243 | 0.1519 | 1 |
|  |  |  |  | GCGTGCAA | 0.182 | 0 | 0.216 | 0.0544 | 0.9314 |
|  |  |  |  | GCGGGCAA | 0.159 | 0.071 | 0.176 | 0.3281 | 1 |
|  |  |  |  | GCATATAG | 0.068 | 0 | 0.081 | 0.2697 | 0.9999 |
|  |  |  |  | ATATGCAA | 0.023 | 0 | 0.027 | 0.5338 | 0.9955 |
| Block 3 (6) | 8385966 | 8793797 | 407.83 | **GTTTTA** | **0.419** | **0.857** | **0.336** | **3.00E-04** | **0.0246** |
|  |  |  |  | GTCTGG | 0.165 | 0.071 | 0.183 | 0.3024 | 0.9999 |
|  |  |  |  | ATTCTG | 0.159 | 0.071 | 0.176 | 0.3281 | 1 |
|  |  |  |  | GCTTGG | 0.141 | 0 | 0.168 | 0.0976 | 0.9778 |
|  |  |  |  | GTTCTG | 0.057 | 0 | 0.068 | 0.3166 | 1 |
|  |  |  |  | GTTTTG | 0.024 | 0 | 0.028 | 0.5229 | 1 |
|  |  |  |  | GCTTTG | 0.018 | 0 | 0.021 | 0.585 | 1 |
|  |  |  |  | GTCTTG | 0.017 | 0 | 0.02 | 0.5926 | 1 |
| Block 4 (8) | 8846442 | 9212034 | 365.59 | **TAACATAC** | **0.341** | **0.857** | **0.244** | **8.98E-06** | **0.0008** |
|  |  |  |  | TGCCGCGT | 0.272 | 0.071 | 0.31 | 0.0658 | 0.9488 |
|  |  |  |  | TGCCGCGC | 0.248 | 0.071 | 0.282 | 0.0946 | 0.9732 |
|  |  |  |  | CGCCGCGC | 0.082 | 0 | 0.097 | 0.2236 | 0.9994 |
|  |  |  |  | CGCTGCAC | 0.021 | 0 | 0.025 | 0.5533 | 1 |
|  |  |  |  | TGCTGCAC | 0.014 | 0 | 0.017 | 0.6259 | 1 |
|  |  |  |  | TAACGCGC | 0.011 | 0 | 0.013 | 0.6656 | 1 |
|  |  |  |  | TAATGCAC | 0.011 | 0 | 0.013 | 0.6705 | 1 |
| Block 5 (2) | 9342079 | 9371042 | 28.96 | CC | 0.759 | 0.929 | 0.727 | 0.1052 | 0.9795 |
|  |  |  |  | TC | 0.15 | 0.071 | 0.165 | 0.3673 | 1 |
|  |  |  |  | CT | 0.071 | 0 | 0.084 | 0.2598 | 0.9999 |
|  |  |  |  | TT | 0.02 | 0 | 0.024 | 0.5596 | 1 |
| Block 6 (4) | 9397350 | 9735624 | 338.27 | **AACT** | **0.487** | **0.929** | **0.403** | **3.00E-04** | **0.0249** |
|  |  |  |  | AATT | 0.127 | 0 | 0.151 | 0.1199 | 0.9867 |
|  |  |  |  | AGTT | 0.125 | 0 | 0.149 | 0.123 | 0.9903 |
|  |  |  |  | GGTG | 0.125 | 0.071 | 0.135 | 0.5086 | 1 |
|  |  |  |  | GGTT | 0.123 | 0 | 0.146 | 0.1262 | 0.9914 |
|  |  |  |  | GGCT | 0.013 | 0 | 0.016 | 0.6367 | 1 |
| Block 7 (4) | 9798399 | 10076303 | 277.90 | CGAC | 0.481 | 0.857 | 0.41 | 0.0022 | 0.1715 |
|  |  |  |  | TAGC | 0.205 | 0.071 | 0.23 | 0.1781 | 0.9981 |
|  |  |  |  | TGAC | 0.186 | 0.071 | 0.207 | 0.2305 | 0.9998 |
|  |  |  |  | TGAT | 0.083 | 0 | 0.098 | 0.2202 | 0.9993 |
|  |  |  |  | TGGT | 0.019 | 0 | 0.023 | 0.5653 | 1 |
|  |  |  |  | CGGC | 0.019 | 0 | 0.022 | 0.5753 | 1 |
| Block 8 (5) | 10283049 | 10429266 | 146.22 | **GTATT** | **0.331** | **0.929** | **0.218** | **2.20E-07** | **<0.0001** |
|  |  |  |  | GTGCT | 0.237 | 0 | 0.282 | 0.0229 | 0.6551 |
|  |  |  |  | GCATT | 0.214 | 0.071 | 0.242 | 0.155 | 0.9955 |
|  |  |  |  | ATGCC | 0.182 | 0 | 0.216 | 0.0544 | 0.9314 |
|  |  |  |  | GCGCT | 0.036 | 0 | 0.042 | 0.4335 | 1 |
| Block 9 (3) | 10490758 | 10532479 | 41.72 | GCG | 0.724 | 1 | 0.672 | 0.0118 | 0.382 |
|  |  |  |  | GCA | 0.151 | 0 | 0.179 | 0.0856 | 0.9657 |
|  |  |  |  | ATG | 0.102 | 0 | 0.122 | 0.1685 | 0.997 |
|  |  |  |  | GTG | 0.014 | 0 | 0.017 | 0.6225 | 1 |
| Block 10 (3) | 10604233 | 10837989 | 233.76 | **CCC** | **0.554** | **1** | **0.469** | **2.00E-04** | **0.0241** |
|  |  |  |  | CTC | 0.208 | 0 | 0.247 | 0.0368 | 0.8368 |
|  |  |  |  | TCC | 0.148 | 0 | 0.176 | 0.0891 | 0.9699 |
|  |  |  |  | TCT | 0.048 | 0 | 0.057 | 0.3583 | 1 |
|  |  |  |  | TTC | 0.042 | 0 | 0.05 | 0.3904 | 1 |
|  |  |  |  |  |  |  |  |  |  |
|  |  |  |  |  |  |  |  |  |  |
|  |  |  |  |  |  |  |  |  |  |
| Block 11 (5) | 10882405 | 10921952 | 39.55 | **ATTAC** | **0.409** | **0.929** | **0.311** | **1.62E-05** | **0.0011** |
|  |  |  |  | GTTGA | 0.25 | 0 | 0.297 | 0.0185 | 0.5552 |
|  |  |  |  | ACTGC | 0.216 | 0 | 0.257 | 0.0323 | 0.7849 |
|  |  |  |  | GTTGC | 0.091 | 0.071 | 0.095 | 0.7822 | 1 |
|  |  |  |  | GCCGC | 0.034 | 0 | 0.041 | 0.4433 | 1 |
| Block 12 (6) | 11028316 | 11334980 | 306.66 | **TAATAT** | **0.349** | **1** | **0.226** | **2.49E-08** | **<0.0001** |
|  |  |  |  | CGCCGC | 0.205 | 0 | 0.243 | 0.0385 | 0.853 |
|  |  |  |  | CGACGC | 0.125 | 0 | 0.149 | 0.123 | 0.9903 |
|  |  |  |  | TAATGT | 0.102 | 0 | 0.122 | 0.1684 | 0.997 |
|  |  |  |  | TAACGC | 0.099 | 0 | 0.118 | 0.1764 | 0.9979 |
|  |  |  |  | TAACGT | 0.083 | 0 | 0.099 | 0.22 | 0.9993 |
|  |  |  |  | TAATGC | 0.023 | 0 | 0.027 | 0.5341 | 1 |
|  |  |  |  | TAATAC | 0.015 | 0 | 0.018 | 0.6178 | 1 |
| Block 13 (2) | 11541978 | 11542053 | 0.08 | CC | 0.705 | 0.929 | 0.662 | 0.0451 | 0.8697 |
|  |  |  |  | CT | 0.261 | 0.071 | 0.297 | 0.0778 | 0.9645 |
|  |  |  |  | TC | 0.034 | 0 | 0.041 | 0.4433 | 1 |
| Block 14 (9) | 11673353 | 11901713 | 228.36 | AAAAGCGGG | 0.306 | 0.143 | 0.337 | 0.148 | 0.9953 |
|  |  |  |  | **AGGAGCGGG** | **0.227** | **0.714** | **0.135** | **2.12E-06** | **0.0003** |
|  |  |  |  | AAGGATAAG | 0.136 | 0 | 0.162 | 0.1057 | 0.98 |
|  |  |  |  | AGAGGTGGG | 0.114 | 0 | 0.135 | 0.144 | 0.9953 |
|  |  |  |  | AAGGGTGGG | 0.08 | 0 | 0.095 | 0.2303 | 0.9998 |
|  |  |  |  | AGAGGTAGG | 0.034 | 0 | 0.041 | 0.4433 | 1 |
|  |  |  |  | AAAAGCGAG | 0.034 | 0.071 | 0.027 | 0.4012 | 1 |
|  |  |  |  | AAAGATAAG | 0.023 | 0 | 0.028 | 0.529 | 1 |
|  |  |  |  | GAAGGTGGA | 0.023 | 0 | 0.027 | 0.5338 | 1 |
|  |  |  |  | AAGAGCGGG | 0.012 | 0.071 | 0.001 | 0.025 | 0.7373 |
|  |  |  |  | AAAGATAGG | 0.011 | 0 | 0.014 | 0.6618 | 1 |
