# Supplementary material for: Genome-Wide Association Study Implicates Testis-Sperm Specific FKBP6 as a Susceptibility Locus for Impaired Acrosome Reaction in Stallions
Source: PLoS Genet. 2012 Dec 20;8(12):e1003139. doi: 10.1371/journal.pgen.1003139 (PMC3527208; doi:10.1371/journal.pgen.1003139)
Supplement: Table S6 — Genes within haplotype blocks defined by confidence interval of LD. Alternative shading is used to easily distinguish between blocks. (DOCX) [file pgen.1003139.s015.docx]

**Table S6.** Genes within haplotype blocks defined by confidence interval of LD. Alternative shading is used to easily distinguish between blocks.

| **Block** | **from (bp)** | **to (bp)** | **Ensembl gene ID** | **gene start (bp)** | **gene end (bp)** | **gene name** |
| --- | --- | --- | --- | --- | --- | --- |
| Block 1 | 8228067 | 8229250 | NONE | NA | NA |  |
| Block 2 | 8977804 | 9183989 | ENSECAG00000014539 | 9008167 | 9020418 |  |
|  |  |  | ENSECAG00000019517 | 9047451 | 9061496 | TRIM56 |
|  |  |  | ENSECAG00000019781 | 9072421 | 9077247 | F7DDC0_HORSE |
|  |  |  | ENSECAG00000010983 | 9086991 | 9092193 | AP1S1 |
|  |  |  | ENSECAG00000018108 | 9094452 | 9096302 |  |
|  |  |  | ENSECAG00000018219 | 9103658 | 9104848 | NAT16 |
|  |  |  | ENSECAG00000018371 | 9131363 | 9133529 | MOGAT3 |
|  |  |  | ENSECAG00000021913 | 9138333 | 9145033 | PLOD3 |
|  |  |  | ENSECAG00000007913 | 9146555 | 9151094 | ZNHIT1 |
|  |  |  | ENSECAG00000011386 | 9156253 | 9160949 | CLDN15 |
|  |  |  | ENSECAG00000013191 | 9163028 | 9166588 | FIS1 |
| Block 3 | 11028316 | 11334980 | ENSECAG00000014578 | 11024597 | 11032198 |  |
|  |  |  | ENSECAG00000017859 | 11037472 | 11048760 | FKBP6 |
|  |  |  | ENSECAG00000022324 | 11115982 | 11117257 |  |
|  |  |  | ENSECAG00000002889 | 11122398 | 11186287 | BAZ1B |
|  |  |  | ENSECAG00000026710 | 11144398 | 11144716 | 7SK |
|  |  |  | ENSECAG00000015512 | 11205656 | 11219991 | BCL7B |
|  |  |  | ENSECAG00000015585 | 11241845 | 11246395 | TBL2 |
|  |  |  | ENSECAG00000021590 | 11252237 | 11269670 | MLXIPL |
|  |  |  | ENSECAG00000009098 | 11295135 | 11297101 | VPS37D |
|  |  |  | ENSECAG00000009728 | 11304566 | 11305262 | DNAJC30 |
|  |  |  | ENSECAG00000012489 | 11305602 | 11316380 | WBSCR22 |
|  |  |  | ENSECAG00000027482 | 11315486 | 11315617 | SCARNA20 |
|  |  |  | ENSECAG00000023810 | 11318505 | 11325902 | F6YNA6_HORSE |
| Block 4 | 11542053 | 11557747 | NONE | NA | NA |  |
| Block 5 | 11758849 | 11770192 | NONE | NA | NA |  |
