# Supplementary material for: Genome-Wide Association Study Implicates Testis-Sperm Specific FKBP6 as a Susceptibility Locus for Impaired Acrosome Reaction in Stallions
Source: PLoS Genet. 2012 Dec 20;8(12):e1003139. doi: 10.1371/journal.pgen.1003139 (PMC3527208; doi:10.1371/journal.pgen.1003139)
Supplement: Table S7 — Genes within blocks defined by solid spine of LD. **genes in bold span more than one haplotype block. Alternative shading is used to easily distinguish between blocks. (DOCX) [file pgen.1003139.s016.docx]

**Table S7:** Genes within blocks defined by solid spine of LD. **genes in bold span more than one haplotype block. Alternative shading is used to easily distinguish between blocks.

| Block | **from (bp)** | **to (bp)** | **Ensembl gene ID** | **gene start (bp)** | **gene end (bp)** | **gene name** |
| --- | --- | --- | --- | --- | --- | --- |
| Block 1 | 8023293 | 8027172 | NONE | NA | NA |  |
| Block 2 | 8228067 | 8385924 | ENSECAG00000002581 | 8248363 | 8264332 |  |
|  |  |  | ENSECAG00000006312 | 8345586 | 8345708 | C7orf59 |
|  |  |  | ENSECAG00000004503 | 8351833 | 8357239 | GAL3ST4 |
|  |  |  | ENSECAG00000008170 | 8358453 | 8364542 | F6TL38_HORSE |
|  |  |  | **ENSECAG00000012736** | **8365528** | **8390178** |  |
| Block 3 | 8385966 | 8793797 | **ENSECAG00000012736** | **8365528** | **8390178** |  |
|  |  |  | ENSECAG00000020068 | 8398542 | 8399883 | PVRIG |
|  |  |  | ENSECAG00000021110 | 8418536 | 8437094 |  |
|  |  |  | ENSECAG00000022363 | 8438059 | 8459535 | ZCWPW1 |
|  |  |  | ENSECAG00000024124 | 8468452 | 8471650 | MEPCE |
|  |  |  | ENSECAG00000006116 | 8472816 | 8473886 | PPP1R35 |
|  |  |  | ENSECAG00000007320 | 8503435 | 8506468 | C7orf61 |
|  |  |  | ENSECAG00000008387 | 8508123 | 8517516 | TSC22D4 |
|  |  |  | ENSECAG00000012983 | 8522801 | 8528928 | NYAP1 |
|  |  |  | ENSECAG00000016244 | 8574815 | 8597177 | AGFG2 |
|  |  |  | ENSECAG00000022553 | 8602807 | 8604418 |  |
|  |  |  | ENSECAG00000023213 | 8605423 | 8606970 | SAP25 |
|  |  |  | ENSECAG00000023227 | 8608654 | 8618458 | LRCH4 |
|  |  |  | ENSECAG00000015517 | 8618783 | 8627667 | FBXO24 |
|  |  |  | ENSECAG00000021766 | 8629026 | 8634028 | PCOLCE |
|  |  |  | ENSECAG00000023837 | 8636523 | 8638579 | MOSPD3 |
|  |  |  | ENSECAG00000024726 | 8642493 | 8655349 | TFR2 |
|  |  |  | ENSECAG00000014917 | 8657022 | 8668432 | F7DMS1_HORSE |
|  |  |  | ENSECAG00000017221 | 8678102 | 8680728 | GNB2 |
|  |  |  | ENSECAG00000017541 | 8683204 | 8690265 | GIGYF1 |
|  |  |  | ENSECAG00000010383 | 8704046 | 8705046 | POP7 |
|  |  |  | ENSECAG00000010733 | 8715477 | 8717341 | EPO |
|  |  |  | ENSECAG00000015672 | 8727123 | 8729320 |  |
|  |  |  | ENSECAG00000016429 | 8732910 | 8760977 |  |
|  |  |  | ENSECAG00000010632 | 8761013 | 8775098 | EPHB4 |
|  |  |  | ENSECAG00000017955 | 8790767 | 8798792 | SLC12A9 |
| Block 4 | 8846442 | 9212034 | ENSECAG00000010102 | 8862310 | 8864810 |  |
|  |  |  | ENSECAG00000011278 | 8924938 | 8950362 | MUC3A |
|  |  |  | ENSECAG00000013954 | 8953790 | 8960989 |  |
|  |  |  | ENSECAG00000014419 | 8974392 | 8974941 |  |
|  |  |  | ENSECAG00000014539 | 9008167 | 9020418 |  |
|  |  |  | ENSECAG00000019517 | 9047451 | 9061496 | TRIM56 |
|  |  |  | ENSECAG00000019781 | 9072421 | 9077247 | F7DDC0_HORSE |
|  |  |  | ENSECAG00000010983 | 9086991 | 9092193 | AP1S1 |
|  |  |  | ENSECAG00000018108 | 9094452 | 9096302 |  |
|  |  |  | ENSECAG00000018219 | 9103658 | 9104848 | NAT16 |
|  |  |  | ENSECAG00000018371 | 9131363 | 9133529 | MOGAT3 |
|  |  |  | ENSECAG00000021913 | 9138333 | 9145033 | PLOD3 |
|  |  |  | ENSECAG00000007913 | 9146555 | 9151094 | ZNHIT1 |
|  |  |  | ENSECAG00000011386 | 9156253 | 9160949 | CLDN15 |
|  |  |  | ENSECAG00000013191 | 9163028 | 9166588 | FIS1 |
|  |  |  | ENSECAG00000022142 | 9211544 | 10389625 | SRRM3 |
| Block 5 | 9342079 | 9371042 | **ENSECAG00000021392** | **9321710** | **9437478** |  |
| Block 6 | 9397350 | 9735624 | **ENSECAG00000021392** | **9321710** | **9437478** |  |
|  |  |  | ENSECAG00000022009 | 9464062 | 9467840 |  |
|  |  |  | ENSECAG00000022094 | 9501337 | 9509709 | MYL10 |
| Block 7 | 9798399 | 10076303 | ENSECAG00000022645 | 9903878 | 10007147 | CUX1 |
|  |  |  | ENSECAG00000024201 | 10021432 | 10032351 | SH2B2 |
|  |  |  | ENSECAG00000005633 | 10066843 | 10091692 | PRKRIP1 |
| Block 8 | 10283049 | 10429266 | ENSECAG00000020258 | 10281426 | 10287887 | SRCRB4D |
|  |  |  | ENSECAG00000006452 | 10329868 | 10330526 | F6ZFH9_HORSE |
|  |  |  | ENSECAG00000020463 | 10346469 | 10347881 | F7E3Y7_HORSE |
| Block 9 | 10490758 | 10532479 | ENSECAG00000019261 | 10456802 | 10508683 | STYXL1 |
|  |  |  | ENSECAG00000024209 | 10511670 | 10515887 |  |
|  |  |  | ENSECAG00000008755 | 10516248 | 10544960 | POR |
| Block10 | 10604233 | 10837989 | ENSECAG00000021173 | 10648090 | 10656087 | RHBDD2 |
|  |  |  | ENSECAG00000026508 | 10708784 | 10708890 | U6 |
|  |  |  | ENSECAG00000022259 | 10728292 | 10729801 | CCL24 |
|  |  |  | ENSECAG00000022659 | 10745930 | 10749290 | CCL26 |
| Block11 | 10882405 | 10921952 | ENSECAG00000022880 | 10895307 | 10931745 | HIP1 |
| Block12 | 11028316 | 11334980 | ENSECAG00000017859 | 11037472 | 11048760 | FKBP6 |
|  |  |  | ENSECAG00000022324 | 11115982 | 11117257 |  |
|  |  |  | ENSECAG00000002889 | 11122398 | 11186287 | BAZ1B |
|  |  |  | ENSECAG00000026710 | 11144398 | 11144716 | 7SK |
|  |  |  | ENSECAG00000015512 | 11205656 | 11219991 | BCL7B |
|  |  |  | ENSECAG00000015585 | 11241845 | 11246395 | TBL2 |
|  |  |  | ENSECAG00000021590 | 11252237 | 11269670 | MLXIPL |
|  |  |  | ENSECAG00000009098 | 11295135 | 11297101 | VPS37D |
|  |  |  | ENSECAG00000009728 | 11304566 | 11305262 | DNAJC30 |
|  |  |  | ENSECAG00000012489 | 11305602 | 11316380 | WBSCR22 |
|  |  |  | ENSECAG00000027482 | 11315486 | 11315617 | SCARNA20 |
|  |  |  | ENSECAG00000023810 | 11318505 | 11325902 | F6YNA6_HORSE |
| Block13 | 11541978 | 11542053 | none | NA | NA |  |
| Block14 | 11673353 | 11901713 | ENSECAG00000017832 | 11699861 | 11707328 | EIF4H |
|  |  |  | ENSECAG00000025936 | 11703520 | 11703600 | eca-mir-590 |
|  |  |  | ENSECAG00000021351 | 11719540 | 11726440 | LAT2 |
|  |  |  | ENSECAG00000000367 | 11730833 | 11753872 | RFC2 |
|  |  |  | ENSECAG00000001107 | 11748405 | 11749229 |  |
|  |  |  | ENSECAG00000002255 | 11751035 | 11752576 |  |
|  |  |  | ENSECAG00000011939 | 11816929 | 11856190 | CLIP2 |
