# Supplementary material for: Genome-Wide Association Study Implicates Testis-Sperm Specific FKBP6 as a Susceptibility Locus for Impaired Acrosome Reaction in Stallions
Source: PLoS Genet. 2012 Dec 20;8(12):e1003139. doi: 10.1371/journal.pgen.1003139 (PMC3527208; doi:10.1371/journal.pgen.1003139)
Supplement: Table S8 — List of horses/equids used for large cohort genotyping of FKBP6 SNPs g.11040315G>A and g.11040379C>A, and association analysis. IAR - impaired acrosome reaction; AR – acrosome reaction; Fertile – horses with confirmed fertility records; Unknown – male horses (stallions and geldings) with no records of fertility or subfertility. (DOCX) [file pgen.1003139.s017.docx]

**Table S8.** List of horses/equids used for large cohort genotyping of *FKBP6* SNPs g.11040315G>A and g.11040379C>A and association analysis. **IAR** - impaired acrosome reaction; **AR** – acrosome reaction; **Fertile** – horses with confirmed fertility records; **Unknown** – male horses (stallions and geldings) with no records of fertility or subfertility.

| **Breed** | **Number of individuals** | **Fertile** | **IAR** | **Normal AR** | **Unknown** |
| --- | --- | --- | --- | --- | --- |
| Akhal-Teke | 2 | 0 | 0 | 0 | 2 |
| Arabian | 6 | 2 | 0 | 0 | 4 |
| Belgian | 4 | 2 | 0 | 0 | 2 |
| Caspian | 5 | 3 | 0 | 0 | 2 |
| Connemara | 4 | 1 | 0 | 0 | 3 |
| Cukurova | 1 | 0 | 0 | 0 | 1 |
| Donkey | 2 | 1 | 0 | 0 | 1 |
| Exmoor | 5 | 3 | 0 | 0 | 2 |
| Friesian | 8 | 1 | 0 | 0 | 7 |
| Icelandic | 1 | 0 | 0 | 0 | 1 |
| Indian Pony | 1 | 0 | 0 | 0 | 1 |
| Miniature | 2 | 0 | 0 | 0 | 2 |
| Missouri Foxtrotter | 1 | 0 | 0 | 0 | 1 |
| Mixed | 2 | 0 | 0 | 0 | 2 |
| Mongolian | 1 | 0 | 0 | 0 | 1 |
| Morgan | 1 | 1 | 0 | 0 | 0 |
| Norwegian Fjord | 2 | 1 | 0 | 0 | 1 |
| Paint | 5 | 1 | 0 | 0 | 4 |
| Polish Primitive | 5 | 0 | 0 | 0 | 5 |
| Quarter Horse | 56 | 35 | 0 | 1 | 20 |
| Tennessee Walking | 1 | 0 | 0 | 0 | 1 |
| Thoroughbred | 145 | 31 | 7 | 4 | 103 |
| Unknown | 5 | 0 | 0 | 0 | 5 |
| **Total** | **265** | **82** | **7** | **5** | **171** |
