# Supplementary material for: Genome-Wide Association Study Implicates Testis-Sperm Specific FKBP6 as a Susceptibility Locus for Impaired Acrosome Reaction in Stallions
Source: PLoS Genet. 2012 Dec 20;8(12):e1003139. doi: 10.1371/journal.pgen.1003139 (PMC3527208; doi:10.1371/journal.pgen.1003139)
Supplement: Table S9 — Allele and genotype frequencies of FKBP6 exon 4 SNPs in GWAS (n = 44) and large (n = 265) study cohorts. (DOCX) [file pgen.1003139.s018.docx]

**Table S9.** Allele and genotype frequencies of *FKBP6* exon 4 SNPs in GWAS (n=44) and large (n=265) study cohorts.

**A.** Allele and genotype frequencies of g.11040315G>A in GWAS horses (n=44; IAR cases = 7; control TBs = 37).

| **SNP**  **g.11040315G>A** | **All frequency (count)** | **Control frequency (count)** | **Case frequency (count)** |
| --- | --- | --- | --- |
| **G** | 0.55 (48) | 0.65 (48) | 0.00 (0) |
| **A** | 0.45 (40) | 0.35 (26) | 1.00 (14) |
|  |  |  |  |
| **GG** | 0.36 (16) | 0.43 (16) | 0.00 (0) |
| **GA** | 0.36 (16) | 0.43 (16) | 0.00 (0) |
| **AA** | 0.27 (12) | 0.14 (5) | 1.00 (7) |

B. Allele and genotype frequencies of g.11040315G>A in all horses (n=265).

| **SNP**  **g.11040315G>A** | **All frequency (count)** | **Fertile frequency (count)** | **IAR frequency (count)** | **Normal AR frequency (count)** | **Unknown frequency (count)** |
| --- | --- | --- | --- | --- | --- |
| **G** | 0.63 (336) | 0.66 (109) | 0.00 (0) | 0.70 (7) | 0.64 (220) |
| **A** | 0.37 (194) | 0.34 (55) | 1.00 (14) | 0.30 (3) | 0.36 (122) |
|  |  |  |  |  |  |
| **GG** | 0.40 (105) | 0.41 (34) | 0.00 (0) | 0.40 (2) | 0.40 (69) |
| **GA** | 0.48 (126) | 0.50 (41) | 0.00 (0) | 0.60 (3) | 0.48 (82) |
| **AA** | 0.13 (34) | 0.09 (7) | 1.00 (7) | 0.00 (0) | 0.12 (20) |

**C.** Allele and genotype frequencies of g.11040379C>A in GWAS horses (n=44; IAR cases = 7; control TBs = 37).

| **SNP2** | **All frequency (count)** | **Control frequency (count)** | **Case frequency (count)** |
| --- | --- | --- | --- |
| **A** | 0.67 (59) | 0.37 (46) | 0.00 (0) |
| **C** | 0.33 (29) | 0.63 (78) | 1.00 (14) |
|  |  |  |  |
| **AA** | 0.43 (19) | 0.32 (12) | 1.00 (7) |
| **AC** | 0.48 (21) | 0.57 (21) | 0.00 (0) |
| **CC** | 0.09 (4) | 0.11 (4) | 0.00 (0) |

**D.** Allele and genotype frequencies of g.11040379C>A in all horses (n=265)

| **SNP2** | **All frequency (count)** | **Fertile frequency (count)** | **IAR frequency (count)** | **Normal AR frequency (count)** | **Unknown frequency (count)** |
| --- | --- | --- | --- | --- | --- |
| **A** | 0.61 (323) | 0.62 (101) | 1.00 (14) | 0.70 (7) | 0.59 (201) |
| **C** | 0.39 (207) | 0.38 (63) | 0.00 (0) | 0.30 (3) | 0.41 (141) |
|  |  |  |  |  |  |
| **AA** | 0.40 (105) | 0.39 (32) | 1.00 (7) | 0.60 (3) | 0.37 (63) |
| **AC** | 0.43 (113) | 0.45 (37) | 0.00 (0) | 0.20 (1) | 0.44 (75) |
| **CC** | 0.18 (47) | 0.16 (13) | 0.00 (0) | 0.20 (1) | 0.19 (33) |

**E.** Combined genotypes at both SNPs

| **Genotype** | **All frequency (count)** | **Fertile frequency (count)** | **IAR frequency (count)** | **Normal AR frequency (count)** | **Unknown frequency (count)** |
| --- | --- | --- | --- | --- | --- |
| AAAA | 0.05 (12) | 0.02 (2) | 1.00 (7) | 0.00 (0) | 0.02 (3) |
| AAAC | 0.05 (13) | 0.04 (3) | 0.00 (0) | 0.00 (0) | 0.06 (10) |
| AACC | 0.03 (9) | 0.02 (2) | 0.00 (0) | 0.00 (0) | 0.04 (7) |
| AGAA | 0.13 (35) | 0.13 (11) | 0.00 (0) | 0.40 (2) | 0.13 (22) |
| AGAC | 0.25 (67) | 0.29 (24) | 0.00 (0) | 0.00 (0) | 0.25 (43) |
| AGCC | 0.09 (24) | 0.07 (6) | 0.00 (0) | 0.20 (1) | 0.1 (17) |
| GGAA | 0.22 (58) | 0.23 (19) | 0.00 (0) | 0.20 (1) | 0.22 (38) |
| GGAC | 0.12 (33) | 0.12 (10) | 0.00 (0) | 0.20 (1) | 0.13 (22) |
| GGCC | 0.05 (14) | 0.06 (5) | 0.00 (0) | 0.00 (0) | 0.05 (9) |
