# Supplementary material for: Genome-Wide Association Study Implicates Testis-Sperm Specific FKBP6 as a Susceptibility Locus for Impaired Acrosome Reaction in Stallions
Source: PLoS Genet. 2012 Dec 20;8(12):e1003139. doi: 10.1371/journal.pgen.1003139 (PMC3527208; doi:10.1371/journal.pgen.1003139)
Supplement: Table S10 — Horse FKBP6 primers for PCR amplification and sequencing of individual exons, and for genotyping and qRT-PCR of exon 4. (DOCX) [file pgen.1003139.s019.docx]

**Table S10**. Horse *FKBP6* primers for PCR amplification and sequencing of individual exons, and for genotyping and qRT-PCR of exon 4.

| **Primer Name** | **Primer sequence 5’-3’** | **Product Size, bp** | **Ta** | **Primer use** |
| --- | --- | --- | --- | --- |
| *FKBP6*.EXON1.F | TACCAGCGGCTGAGTCAGAG | 67 | 58^0^C | Sequencing |
| *FKBP6*.EXON1.R | GGATGACGTCCTTCAGCA |  |  |  |
| *FKBP6*.EXON2.F | TTCTGGCTATCTGGAGCACA | 72 | 58^0^C |  |
| *FKBP6*.EXON2.R | TTCATGAGCCGAGGAGTCTT |  |  |  |
| *FKBP6*.EXON3.F | TATTACCCTTTGGGGCATGG | 199 | 58^0^C |  |
| *FKBP6*.EXON3.R | CGAGAGGGCACAGAACTTGT |  |  |  |
| *FKBP6*.EXON4.F | CAAGATCAATTTCCGCTTCA | 98 | 58^0^C |  |
| *FKBP6*.EXON4.R | TTGGCGTCATAGAAACGATG |  |  |  |
| *FKBP6*.EXON5.F | CCTTGTTGCTTCTCCATCG | 194 | 58^0^C |  |
| *FKBP6*.EXON5.F | CTGTCCACAGCGAAAGAGG |  |  |  |
| *FKBP6*.EXON6.F | GCCTGTCTCCTGATGACTGAG | 110 | 58^0^C |  |
| *FKBP6*.EXON6.R | CTGGCCAGTTTCTTCAGCTC |  |  |  |
| *FKBP6*.EXON5-6.F | CCTTGTTGCTTCTCCATCG | 382 gDNA; 279 cDNA | 58^0^C |  |
| *FKBP6*.EXON5-6.R | GTCATGATTGAAGGGCTGTG |  |  |  |
| *FKBP6*.EXON7.F | CTGTTACAAGGACTACACGGATAAA | 90 | 58^0^C |  |
| *FKBP6*.EXON7.R | CAGTTTTCTCCTACTGCAGAGTCA |  |  |  |
| *FKBP6.EX4.SNaPShot G-A (R)* | CTTTCAGGACCTTCTGAAG |  | 58^0^C | Genotyping |
| *FKBP6.EX4.SNaPShot C-A (F)* | GGCAACTACCTTTTCCGCCAA |  |  |  |
| *FKBP6.EXON4.F* | CAATTTCCGCTTCAGAAGGT | 98 | 58^0^C | qRT-PCR |
| FKBP6.EXON4.R | ATACCTCACTTTGGCGTCAT |  |  |  |
