# Supplementary material for: Genome-Wide Association Study Implicates Testis-Sperm Specific FKBP6 as a Susceptibility Locus for Impaired Acrosome Reaction in Stallions
Source: PLoS Genet. 2012 Dec 20;8(12):e1003139. doi: 10.1371/journal.pgen.1003139 (PMC3527208; doi:10.1371/journal.pgen.1003139)
Supplement: Table S11 — FKBP6 exon primers for the discovery of splice variants: expected and observed PCR amplicons in testis and sperm. (DOCX) [file pgen.1003139.s020.docx]

**Table S11.** *FKBP6* exon (intron-spanning) primers for the discovery of splice variants with expected and observed PCR amplicons in testis and sperm; *PRM2*, *SPA17* and *PTPRC* are control genes for RNA quality.

| **Primer Name** | **Primer Sequence 5’-3’** | **Expected amplicon** | **Observed**  **amplicon** | **Normal Sperm** | **IAR Sperm** | **Testis** |
| --- | --- | --- | --- | --- | --- | --- |
| EXON 1&2.F | CTGAGTCAGAGGATGCTGGA | 182bp | 182bp | **-** | **-** | **+** |
| EXON 1&2.R | TTCATGAGCCGAGGAGTCTT |  |  |  |  |  |
| EXON 1&3.F | GAGTCAGAGGATGCTGGACA | 336bp | 336bp | **-** | **-** | **+** |
| EXON 1&3.R | ATCTCGAACAGAACGGTGGT |  |  |  |  |  |
| EXON 1&4.F | CTGAGTCAGAGGATGCTGGA | 502bp | 502bp; 297bp  (no exon 3) | **-** | **-** |  |
| EXON 1&4.R | CTTTGGCGTCATAGAAACGA |  |  |  |  |  |
| EXON 1&5.F | CTGAAGGACGTCATCCGAGA | 646bp | 646bp; 443bp  (no exon 3) | **-** | **-** | **+** |
| EXON 1&5.R | TGGCATTCTTCTGGTCAATG |  |  |  |  |  |
| EXON 1&6.F | TACCAGCGGCTGAGTCAGAG | 828bp | 828bp; 625bp  (no exon 3) | **-** | **-** | **+** |
| EXON 1&6.R | GGCCAGTTTCTTCAGCTCAT |  |  |  |  |  |
| EXON 1&7.F | CTGAGTCAGAGGATGCTGGA | 885bp | 885bp; 682bp  (no exon 3) | **-** | **-** | **+** |
| EXON 1&7.R | ATCACAAGGGGCAAACATTC |  |  |  |  |  |
| EXON 2&3.F | GAGCACATGGACAAGCCTTT | 201bp | 201bp | **+** | **+** | **+** |
| EXON 2&3.R | TGGTGTTTGGGGGTATCAAC |  |  |  |  |  |
| EXON 2&4.F | TTCTGGCTATCTGGAGCACA | 392bp | 392bp, 189bp  (no exon 3) | **+** | **+** | **+** |
| EXON 2&4.R | CTTTGGCGTCATAGAAACGA |  |  |  |  |  |
| EXON 2&5.F | AGGAAGACTCCTCGGCTCAT | 529bp | 529bp | **+** | **+** | **+** |
| EXON 2&5.R | TGGCATTCTTCTGGTCAATG |  |  |  |  |  |
| EXON 2&6.F | TTCTGGCTATCTGGAGCACA | 711bp | 711bp; 508bp  (no exon 3) | **+** | **+** | **+** |
| EXON 2&6.R | CTGGCCAGTTTCTTCAGCTC |  |  |  |  |  |
| EXON 2&7.F | TTCTGGCTATCTGGAGCACA | 775bp | 775bp; 572bp  (no exon 3); 147bp (no exons 3,4,5,6) | **+** | **+** | **+** |
| EXON 2&7.R | ATCACAAGGGGCAAACATTC |  |  |  |  |  |
| EXON 3&4.F | CTGGCCAGGTTTCTGTTCA | 196bp | 196bp | **+** | **+** | **+** |
| EXON 3&4.R | CTGCCACTTTCAGGACCTTC |  |  |  |  |  |
| EXON 3&5.F | ACAAGTTCTGTGCCCTCTCG | 315bp | 315bp | **+** | **+** | **+** |
| EXON 3&5.R | TGGCATTCTTCTGGTCAATG |  |  |  |  |  |
| EXON 3&6.F | TATTACCCTTTGGGGCATGG | 627bp | 627bp | **+** | **+** | **+** |
| EXON 3&6.R | CTGGCCAGTTTCTTCAGCTC |  |  |  |  |  |
| EXON 3&7.F | TATTACCCTTTGGGGCATGG | 677bp | 677bp | **+** | **+** | **+** |
| EXON 3&7.R | ACATTCGGTGGCACATTTCT |  |  |  |  |  |
| EXON 4&5.F | TGAGGTATAAACGGGCCTTG | 186bp | 186bp | **+** | **+** | **+** |
| EXON 4&5.R | TGGCATTCTTCTGGTCAATG |  |  |  |  |  |
| EXON 4&6.F | GGTCCTGAAAGTGGCAGCTA | 396bp | 396bp | **+** | **+** | **+** |
| EXON 4&6.R | GGCCAGTTTCTTCAGCTCAT |  |  |  |  |  |
| EXON 4&7.F | GGTCCTGAAAGTGGCAGCTA | 460bp | 460bp | **+** | **+** | **+** |
| EXON 4&7.R | ATCACAAGGGGCAAACATTC |  |  |  |  |  |
| EXON 5&6.F | GCCAAGCTTCTTGTTCTCCT | 196bp | 196bp | **+** | **+** | **+** |
| EXON 5&6.R | CCTTCTGGGCTTGGACTAGA |  |  |  |  |  |
| EXON 5&7.F | GCCAAGCTTCTTGTTCTCCT | 309bp | 309bp | **+** | **+** | **+** |
| EXON 5&7.R | ATCACAAGGGGCAAACATTC |  |  |  |  |  |
| EXON 6&7.F | GCCTGTCTCCTGATGACTGA | 174bp | 174bp | **+** | **+** | **+** |
| EXON 6&7.R | ATCACAAGGGGCAAACATTC |  |  |  |  |  |
| *PRM2* 1&2.F | CGGGAGCTACTACCGCTACA | 167bp | 351bp | **+** | **+** | **+** |
| *PRM2* 1&2.R | GCCTTCTGCATCTTCTCCTC |  |  |  |  |  |
| *SPA17* 3&4.F | CAGAATGGGGAGCTAAGGTAGA | 104 bp | 104 bp | **+** | **+** | **+** |
| *SPA17* 3&4.R | TGTCTGCGACTTTTCTTTGC |  |  |  |  |  |
| *SPA17* 4&5.F | GAATCACCTGAGAAATGTGAGC | 154 | 154 bp | **+** | **+** | **+** |
| *SPA17* 4&5.R | CTAAGTGTCCCGGAAAGCT |  |  |  |  |  |
| *SPA17* 3&5.F | CAGAATGGGGAGCTAAGGTAGA | 213 | 213 bp | **+** | **+** | **+** |
| *SPA17* 3&5.R | CTAAGTGTCCCGGAAAGCT |  |  |  |  |  |
| *PTPRC*.F | AATTTGACCCTGCATCTCCA | 147bp | 147 bp | **-** | **-** | **+** |
| *PTPRC*.R | ATTCCACGGGTGTTCAGC |  |  |  |  |  |
