# Supplementary material for: Genome-Wide Association Study Implicates Testis-Sperm Specific FKBP6 as a Susceptibility Locus for Impaired Acrosome Reaction in Stallions
Source: PLoS Genet. 2012 Dec 20;8(12):e1003139. doi: 10.1371/journal.pgen.1003139 (PMC3527208; doi:10.1371/journal.pgen.1003139)
Supplement: Table S12 — Equine FKBP6 exon 4 cDNA sequencing: monoallelic expression in testis and sperm. (DOCX) [file pgen.1003139.s021.docx]

**Table S12.** Equine *FKBP6* exon 4 cDNA sequencing: monoallelic expression in testis and sperm.

| **Stallion** | **Code** | **gDNA**  **A>G** | **cDNA A>G**  **(Testis)** | **cDNA A>G**  **(Sperm)** | **gDNA A>C** | **cDNA A>C**  **(Testis)** | **cDNA A>C**  **(Sperm)** |
| --- | --- | --- | --- | --- | --- | --- | --- |
| IAR case | HS03 | AA | AA | AA | AA | AA | AA |
| Control 1 | H281 | AG | GG | n/a | AC | CC | n/a |
| Control 2 | H343 | AG | n/a | GG | AC | n/a | Noisy |
| Control 3 | H347 | AG | GG | GG | AC | CC | CC |
| Control 4 | H356 | AG | GG | GG | AC | CC | CC |
| Control 5 | H410 | AG | n/a | GG | AC | n/a | CC |
| Control 6 | H452 | AG | GG | GG | AC | CC | CC |
| Control 7 | H470 | AG | GG | n/a | AC | CC | n/a |
| Control 8 | H474 | AG | GG | n/a | AC | CC | n/a |
