# Supplementary material for: Genome-Wide Association Study Implicates Testis-Sperm Specific FKBP6 as a Susceptibility Locus for Impaired Acrosome Reaction in Stallions
Source: PLoS Genet. 2012 Dec 20;8(12):e1003139. doi: 10.1371/journal.pgen.1003139 (PMC3527208; doi:10.1371/journal.pgen.1003139)

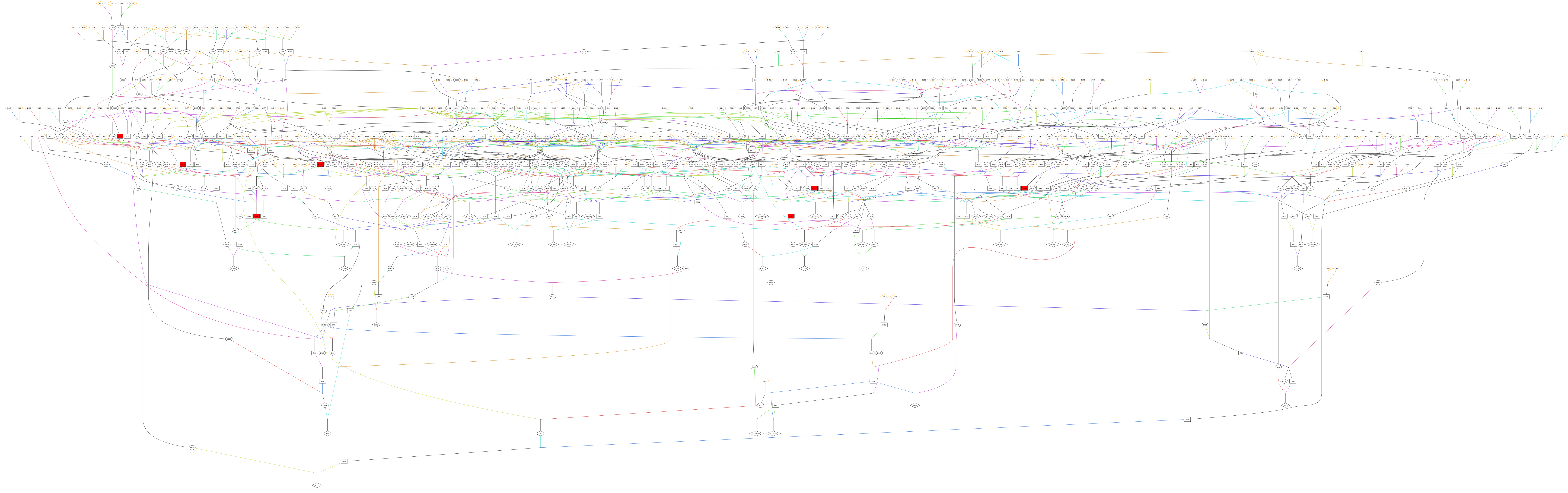

Supplement: Figure S1 — Pedigree analysis. Pedigree of the 44 Thoroughbred horses used for GWAS in this study. Rectangles indicate males, ovals indicate females, diamonds indicate the 37 controls and red highlights indicate the 7 affected stallions. The pedigree has been generated in PEDIGRAPH software. (PDF) [file pgen.1003139.s001.pdf]
